# Deep learning models for acute kidney injury prediction: multi-center external validation and evaluation under simulated continuous monitoring conditions

**DOI:** 10.1038/s41746-026-02722-2

**Published:** 2026-05-08

**Authors:** Kyung Hyun Lee, Donghwee Yoon, Hyunsun Lim, Ki-Byung Lee, Yong Kyu Lee

**Affiliations:** 1 AITRICS Co., Ltd., Seoul, 06221 Republic of Korea; 2 Department of Digital Health, SAIHST, Sungkyunkwan University, Seoul, 06355 Korea; 3 Department of Research and Analysis, National Health Insurance Service Ilsan Hospital, Goyangshi, Gyeonggi–do Republic of Korea; 4 Division of Pulmonary, Allergy and Critical Care Medicine, Department of Internal Medicine, Chuncheon Sacred Heart Hospital, Hallym University Medical Center, Chuncheon, Gangwon-do Republic of Korea; 5 Department of Internal Medicine, Nephrology Subdivision, National Health Insurance Service Ilsan Hospital, Goyangshi, Gyeonggi–do Republic of Korea

**Keywords:** Computational biology and bioinformatics, Diseases, Health care, Mathematics and computing, Medical research, Risk factors

## Abstract

Acute kidney injury (AKI) is a common hospital complication with substantial morbidity and mortality. Deep learning models for AKI prediction show strong development-cohort performance, but single-point evaluation fails to capture behaviour under continuous monitoring. We conducted a multi-centre retrospective study using electronic health records from three cohorts (*n* = 157,323 admissions): National Health Insurance Service Ilsan Hospital (development), Chuncheon Sacred Heart Hospital, and MIMIC-IV (external validation). Three deep learning architectures (LSTM-Attention, Masked CNN, ITE-Transformer) and two baselines (XGBoost, logistic regression) were developed across 0-, 48-, and 72-h horizons, with an online simulation framework generating predictions at 12-h intervals before onset. Deep learning substantially outperformed baselines externally (AUROC 0.956–0.963 vs. 0.630–0.686). Online simulation revealed that 0-h models exhibited “clinical faithfulness”—consistent AUROC improvement as onset approached (Mann–Kendall significant in 15/15 combinations)—whereas longer horizons showed unstable trajectories. Notably, the highest single-point AUROC model (Masked CNN, 0.961) had the worst deployment profile (NNE 17.6–564), while ITE-Transformer (AUROC 0.924) achieved the most favourable alert burden (NNE 1.5–2.4). Deployment-oriented evaluation should complement conventional metrics for continuous monitoring models.

## Introduction

Acute kidney injury (AKI) is a common and serious complication of hospitalisation, affecting approximately 10–20% of hospitalised adults and more than 50% of patients admitted to intensive care units^1,2^. The condition is associated with increased in-hospital mortality, greater risk of progression to chronic kidney disease, and major adverse cardiovascular events^3^. Growing evidence suggests that early recognition and timely intervention may improve outcomes in patients at risk of developing AKI, appealing for the clinical importance of accurate prediction tools^4^.

Over the past decade, considerable efforts have been devoted to developing prediction models for AKI, ranging from traditional logistic regression approaches to more recent deep learning architectures^5–9^. Several models have demonstrated strong discriminative performance, with AUROC values exceeding 0.90 in development settings^8,10,11^. Despite these encouraging results, systematic reviews have consistently highlighted barriers to clinical translation, including methodological heterogeneity, limited external validation, and inadequate calibration assessment^12,13^. A recent multi-national study on continuous AKI prediction further emphasised that most existing models lack evaluation under realistic deployment conditions^5^.

An additional, and perhaps more fundamental, concern relates to how prediction models are evaluated, which disaccords with how they would be used in clinical practice. The vast majority of existing studies rely on single-point evaluation, in which model performance is assessed at a fixed prediction horizon. In clinical practice, however, early warning systems are designed to operate continuously, generating updated risk assessments as new patient data becomes available—much like the National Early Warning Score (NEWS)^14^. A model that achieves high discrimination at a fixed 48-h horizon may not necessarily perform well when predictions are generated repeatedly throughout the course of hospitalisation. This gap between evaluation methodology and deployment reality represents a core challenge for clinical informatics, as ICU clinicians frequently report alert fatigue from clinical decision support systems, with alert specificity and temporal relevance identified as key determinants of appropriate clinician response^15,16^.

This evaluation gap may have important clinical implications. Models optimised for single-point performance could potentially exhibit less stable temporal behaviour during deployment, generating inconsistent alerts that may contribute to alert fatigue or delayed recognition of clinical deterioration. We hypothesised that the temporal dynamics of AKI—in which significant renal insult is estimated to begin approximately 48 h before the clinical diagnosis of AKI is established^17–19^—might influence model performance across prediction horizons during continuous monitoring.

It is important to note that prediction models developed on historical data inherently adopt an observational framework, and the gap between prediction and actionable clinical decision-making—which requires causal considerations—remains an important challenge for the field. Models trained to predict AKI from retrospective data do not account for how clinician behaviour might change in response to model outputs, and this distinction between prediction and intervention warrants careful consideration when evaluating clinical utility.

To address this evaluation gap, we developed and externally validated deep learning models for AKI prediction across three independent hospital settings. In addition, we propose an online simulation framework designed to evaluate model performance under conditions that replicate continuous clinical monitoring. We compared three deep learning architectures representing the major families of approaches for clinical time-series—recurrent (LSTM-Attention), convolutional (Masked CNN), and attention-based (ITE-Transformer)—alongside two non-deep-learning baselines (XGBoost and logistic regression). These models were evaluated across three prediction horizons selected to align with AKI pathophysiology: 0-h (capturing the complete clinical cascade), 48-h (coinciding with the estimated onset of significant renal insult^17–19^, and specifically examining whether algorithmic prediction could precede the typical timing of clinical recognition), and 72-h (preceding detectable injury). We introduced the concept of ‘clinical faithfulness’—defined as consistent improvement of model confidence as the clinical process of AKI progresses—as a criterion for evaluating the deployment suitability of continuous monitoring models.

## Results

### Patient characteristics

The study included 157,323 admissions across three sites. The incidence of AKI was 11.42% at NHIS, 10.66% at CSHH, and 4.86% at MIMIC-IV. The lower AKI incidence at MIMIC-IV, despite the ICU-only setting, reflects the uniform application of temporal data requirements across all cohorts. Our inclusion criteria required a minimum observation period of 12 h for all patients, and AKI patients additionally required at least 72 h of pre-event data for model training and evaluation. These requirements disproportionately excluded ICU patients who developed AKI early in their admission—a population in which AKI incidence is known to be high^1^—resulting in a selected cohort with a lower apparent incidence (Supplementary Fig. S1). Baseline patient characteristics are presented in Table 1 and Supplementary Table S1.

**Table 1:** Baseline characteristics of the study population

Variable	Category	NHIS	CSHH	MIMIC-IV	*P* value
*n*		83 085	68 615	5623	
Age		59.7 (17.4)	56.9 (19.7)	60.3 (16.4)	<0.001
Sex	Male	38 875 (46.8)	36 436 (53.1)	3326 (59.1)	<0.001
	Female	44 210 (53.2)	32 179 (46.9)	2297 (40.9)	
BMI		23.7 (3.8)	23.6 (3.9)	26.6 (6.6)	<0.001
Length of stay		4.6 [2.8, 9.0]	4.0 [2.0, 7.0]	1.6 [1.2, 2.5]	<0.001
Systolic blood pressure		131.8 (20.7)	123.2 (20.1)	123.5 (24.8)	<0.001
Diastolic blood pressure		77.6 (13.0)	74.7 (11.6)	69.4 (17.9)	<0.001
Heart rate		82.7 (15.9)	82.3 (15.8)	88.4 (20.0)	<0.001
Respiratory rate		19.0 (2.1)	20.2 (2.0)	19.1 (5.9)	<0.001
Oxygen saturation		96.6 (3.1)	96.8 (2.7)	97.3 (3.4)	<0.001
Temperature		36.8 (0.5)	36.8 (0.6)	36.7 (0.8)	<0.001
Creatinine		0.8 [0.6, 1.0]	0.9 [0.7, 1.0]	0.9 [0.7, 1.5]	<0.001
AKI incidence	Yes	9485 (11.4)	7321 (10.7)	273 (4.9)	<0.001
	No	73 600 (88.6)	61 294 (89.3)	5350 (95.1)	
AKI stage	Stage 1	8875 (10.7)	6802 (9.9)	213 (3.8)	<0.001
	Stage 2	571 (0.7)	474 (0.7)	18 (0.3)	
	Stage 3	39 (0.0)	45 (0.1)	42 (0.7)	

Data are presented as *n* (%), mean (SD), or median [IQR]. *P* values were calculated using one-way ANOVA for normally distributed continuous variables, Kruskal–Wallis test for skewed continuous variables, and chi-squared test for categorical variables. The NHIS cohort was used for model development and internal validation; CSHH and MIMIC-IV served as independent external validation cohorts. AKI was defined and staged according to KDIGO serum creatinine criteria.

*AKI* acute kidney injury, *BMI* body mass index, *CSHH* Chuncheon Sacred Heart Hospital, *MIMIC-IV* Medical Information Mart for Intensive Care IV, *NHIS* National Health Insurance Service Ilsan Hospital.

### Single-point performance (Development)

**Fig. 1:**
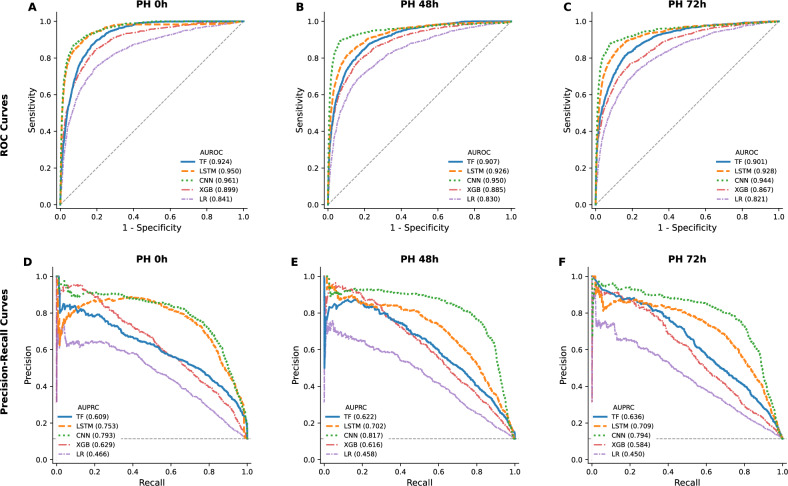
Discrimination performance in the development cohort (NHIS). Receiver operating characteristic (ROC) curves (**A**–**C**) and precision–recall curves (**D**–**F**) for all five models across three prediction horizons: 0 h (**A**, **D**), 48 h (B, E), and 72 h (**C**, **F**). Five model architectures are shown: ITE-Transformer (TF; blue solid), LSTM-Attention (LSTM; orange dashed), Masked CNN (CNN; green dotted), XGBoost (XGB; red dash-dot), and Logistic Regression (LR; purple dash-dot-dot). AUROC and AUPRC values are reported in each panel legend. The Masked CNN achieved the highest single-point AUROC (0.961) at 0 h prediction. The diagonal dashed line in ROC plots indicates random classifier performance.

In internal validation, all five models demonstrated discrimination ranging from AUROC 0.82–0.96 (Table 2 and Fig. 1). Among deep learning architectures, Masked CNN achieved the highest single-point AUROC (0.961) at 0 h prediction. Baseline models achieved lower but reasonable discrimination (XGBoost: 0.899; logistic regression: 0.841 at 0 h). Performance decreased with longer prediction horizons across all model types. Comprehensive single-point results are provided in Supplementary Table S2.

**Table 2:** Internal validation performance at NHIS (development cohort)

Model	PH	AUROC (95% CI)	AUPRC (95% CI)	Sens, % (95% CI)	Spec, % (95% CI)	PPV, % (95% CI)	NPV, % (95% CI)
**LSTM-attention**	0 h	0.950 (0.941–0.958)	0.753 (0.722–0.785)	89.8 (87.9–91.7)	87.6 (86.8–88.3)	48.2 (45.7–50.4)	98.5 (98.2–98.8)
	48 h	0.926 (0.916–0.935)	0.702 (0.671–0.730)	81.6 (79.0–83.8)	89.6 (88.9–90.3)	50.3 (47.6–52.8)	97.4 (97.0–97.8)
	72 h	0.928 (0.918–0.938)	0.709 (0.678–0.739)	85.0 (82.6–87.2)	87.9 (87.1–88.6)	47.4 (45.1–49.9)	97.9 (97.5–98.2)
**Masked CNN**	0 h	0.961 (0.955–0.967)	0.793 (0.764–0.822)	86.9 (84.8–88.9)	92.8 (92.2–93.4)	61.0 (58.4–63.6)	98.2 (97.9–98.5)
	48 h	0.950 (0.941–0.960)	0.817 (0.791–0.844)	89.4 (87.3–91.3)	93.4 (92.8–93.9)	63.5 (60.8–66.0)	98.6 (98.3–98.8)
	72 h	0.944 (0.934–0.953)	0.794 (0.767–0.820)	87.8 (85.9–89.7)	92.2 (91.6–92.7)	59.1 (56.5–61.6)	98.3 (98.0–98.6)
**ITE-transformer**	0 h	0.924 (0.916–0.931)	0.609 (0.576–0.644)	87.0 (84.9–89.2)	82.9 (82.0–83.8)	39.6 (37.3–41.8)	98.0 (97.6–98.4)
	48 h	0.907 (0.897–0.915)	0.622 (0.589–0.655)	84.3 (81.9–86.5)	81.1 (80.2–82.0)	36.5 (34.4–38.6)	97.6 (97.2–97.9)
	72 h	0.901 (0.890–0.911)	0.636 (0.601–0.666)	81.1 (78.6–83.5)	84.1 (83.3–85.0)	39.8 (37.3–41.9)	97.2 (96.8–97.6)
**XGBoost**	0 h	0.899 (0.888–0.910)	0.629 (0.594–0.662)	85.0 (82.7–87.3)	80.4 (79.4–81.3)	35.9 (33.7–37.8)	97.7 (97.3–98.0)
	48 h	0.885 (0.873–0.897)	0.616 (0.582–0.649)	79.3 (76.8–81.9)	82.7 (81.8–83.6)	37.1 (34.8–39.3)	96.9 (96.4–97.3)
	72 h	0.867 (0.854–0.880)	0.584 (0.549–0.619)	76.7 (74.1–79.4)	81.4 (80.4–82.3)	34.7 (32.5–36.8)	96.4 (96.0–96.9)
**Logistic regression**	0 h	0.841 (0.825–0.855)	0.466 (0.431–0.502)	75.6 (72.8–78.4)	80.3 (79.3–81.2)	33.0 (31.1–35.2)	96.2 (95.7–96.7)
	48 h	0.830 (0.815–0.844)	0.458 (0.422–0.494)	68.5 (65.3–71.5)	84.0 (83.1–84.8)	35.5 (33.2–37.8)	95.4 (94.8–95.9)
	72 h	0.821 (0.806–0.836)	0.450 (0.416–0.486)	72.1 (69.4–74.9)	78.5 (77.4–79.4)	30.1 (28.2–32.1)	95.6 (95.1–96.1)

Internal validation at NHIS (*n* = 8309; AKI prevalence 11.4%). 95% CIs from bootstrap resampling (*n* = 1000). Sensitivity and specificity computed at optimal thresholds determined from the development set (LSTM-Attention: 0.817/0.801/0.724; Masked CNN: 0.622/0.548/0.532; ITE-Transformer: 0.789/0.694/0.677; XGBoost: 0.325/0.377/0.376; Logistic Regression: 0.495/0.555/0.506 for PH 0 h/48 h/72 h, respectively).

*PH* prediction horizon, *AUROC* area under the receiver operating characteristic curve, *AUPRC* area under the precision–recall curve, *Sens* sensitivity, Spec specificity, *PPV* positive predictive value, *NPV* negative predictive value.

### External validation performance

External validation revealed heterogeneous performance across sites (Tables 3–4 and Supplementary Fig. S2). At CSHH, the ITE-Transformer (0 h) achieved an AUROC of 0.912 compared with 0.818 for the Masked CNN. At MIMIC-IV, deep learning models maintained strong discrimination at the 0 h horizon (AUROC 0.956–0.963), substantially outperforming baseline models (XGBoost: 0.686; logistic regression: 0.630). All model types showed substantial degradation at the 72 h horizon. The performance gap between deep learning and baseline models was most pronounced at external sites, suggesting that the temporal modelling capabilities of deep learning architectures provide greater generalisability advantage.

**Table 3:** External validation performance at CSHH

Model	PH	AUROC (95% CI)	AUPRC (95% CI)	Sens, % (95% CI)	Spec, % (95% CI)	PPV, % (95% CI)	NPV, % (95% CI)
**LSTM-attention**	0 h	0.864 (0.860–0.869)	0.428 (0.416–0.440)	76.1 (75.0–77.0)	80.5 (80.2–80.8)	31.8 (31.1–32.5)	96.6 (96.4–96.7)
	48 h	0.761 (0.755–0.767)	0.318 (0.307–0.328)	51.2 (50.1–52.3)	85.2 (85.0–85.5)	29.3 (28.5–30.0)	93.6 (93.4–93.8)
	72 h	0.631 (0.623–0.639)	0.208 (0.201–0.217)	65.1 (63.9–66.2)	53.0 (52.6–53.3)	14.2 (13.8–14.5)	92.7 (92.4–93.0)
**Masked CNN**	0 h	0.818 (0.814–0.823)	0.365 (0.353–0.377)	72.8 (71.8–73.9)	72.5 (72.2–72.8)	24.0 (23.5–24.6)	95.7 (95.5–95.9)
	48 h	0.793 (0.787–0.799)	0.389 (0.377–0.401)	69.2 (68.2–70.4)	76.2 (75.8–76.5)	25.7 (25.1–26.4)	95.4 (95.2–95.6)
	72 h	0.759 (0.753–0.766)	0.317 (0.307–0.328)	71.3 (70.2–72.4)	65.1 (64.7–65.5)	19.6 (19.1–20.1)	95.0 (94.8–95.2)
**ITE-transformer**	0 h	0.912 (0.909–0.914)	0.553 (0.541–0.565)	78.7 (77.7–79.7)	84.9 (84.6–85.2)	38.4 (37.7–39.2)	97.1 (96.9–97.3)
	48 h	0.822 (0.816–0.827)	0.430 (0.418–0.442)	74.0 (73.0–75.0)	75.8 (75.4–76.1)	26.7 (26.1–27.3)	96.1 (95.9–96.2)
	72 h	0.776 (0.771–0.782)	0.316 (0.305–0.327)	69.4 (68.3–70.4)	72.0 (71.7–72.4)	22.9 (22.3–23.4)	95.2 (95.0–95.3)
**XGBoost**	0 h	0.824 (0.819–0.829)	0.404 (0.392–0.416)	74.6 (73.6–75.6)	75.6 (75.2–76.0)	26.7 (26.1–27.3)	96.1 (96.0–96.3)
	48 h	0.806 (0.801–0.811)	0.387 (0.376–0.399)	66.7 (65.6–67.7)	79.3 (79.0–79.6)	27.8 (27.1–28.4)	95.2 (95.0–95.4)
	72 h	0.799 (0.793–0.805)	0.383 (0.372–0.396)	65.2 (64.1–66.2)	80.5 (80.2–80.8)	28.5 (27.8–29.2)	95.1 (94.9–95.3)
**Logistic regression**	0 h	0.796 (0.791–0.802)	0.351 (0.339–0.363)	67.2 (66.1–68.1)	78.3 (77.9–78.6)	27.0 (26.3–27.6)	95.2 (95.0–95.4)
	48 h	0.777 (0.771–0.782)	0.321 (0.310–0.333)	57.2 (56.1–58.2)	82.6 (82.3–82.9)	28.2 (27.5–28.9)	94.2 (94.0–94.4)
	72 h	0.779 (0.773–0.784)	0.320 (0.310–0.331)	64.8 (63.7–65.8)	77.1 (76.7–77.4)	25.2 (24.6–25.8)	94.8 (94.6–95.0)

External validation at CSHH (*n* = 68,615; AKI prevalence 10.7%). Thresholds determined from the development set (LSTM-Attention: 0.817/0.801/0.724; Masked CNN: 0.622/0.548/0.532; ITE-Transformer: 0.789/0.694/0.677; XGBoost: 0.325/0.377/0.376; Logistic Regression: 0.495/0.555/0.506 for PH 0 h/48 h/72 h). Abbreviations as in Table 2.

**Table 4:** External validation performance at MIMIC-IV

Model	PH	AUROC (95% CI)	AUPRC (95% CI)	Sens, % (95% CI)	Spec, % (95% CI)	PPV, % (95% CI)	NPV, % (95% CI)
**LSTM-attention**	0 h	0.956 (0.948–0.963)	0.485 (0.423–0.547)	99.3 (98.1–100.0)	87.4 (86.4–88.3)	28.6 (25.6–31.4)	100.0 (99.9–100.0)
	48 h	0.851 (0.838–0.864)	0.143 (0.125–0.165)	97.1 (95.1–98.8)	65.1 (63.8–66.3)	12.4 (11.0–13.9)	99.8 (99.6–99.9)
	72 h	0.599 (0.564–0.632)	0.070 (0.059–0.087)	96.0 (93.4–98.0)	11.8 (11.0–12.6)	5.3 (4.6–5.9)	98.3 (97.2–99.2)
**Masked CNN**	0 h	0.963 (0.957–0.967)	0.420 (0.370–0.475)	99.3 (98.1–100.0)	89.7 (88.8–90.5)	32.9 (29.5–36.0)	100.0 (99.9–100.0)
	48 h	0.877 (0.861–0.893)	0.202 (0.174–0.238)	96.0 (93.5–98.1)	69.7 (68.4–70.9)	13.9 (12.3–15.5)	99.7 (99.5–99.9)
	72 h	0.648 (0.620–0.678)	0.082 (0.065–0.104)	90.1 (86.6–93.5)	29.0 (27.7–30.2)	6.1 (5.4–6.8)	98.3 (97.6–98.9)
**ITE-transformer**	0 h	0.962 (0.956–0.968)	0.487 (0.426–0.553)	99.6 (98.8–100.0)	80.7 (79.6–81.7)	20.9 (18.5–23.1)	100.0 (99.9–100.0)
	48 h	0.900 (0.889–0.911)	0.244 (0.210–0.290)	98.5 (96.8–99.7)	68.0 (66.7–69.2)	13.6 (12.0–15.1)	99.9 (99.8–100.0)
	72 h	0.713 (0.682–0.743)	0.109 (0.091–0.133)	98.2 (96.4–99.6)	7.4 (6.6–8.1)	5.1 (4.5–5.7)	98.7 (97.5–99.7)
**XGBoost**	0 h	0.686 (0.654–0.716)	0.111 (0.090–0.140)	94.9 (92.3–97.3)	16.6 (15.7–17.6)	5.5 (4.8–6.1)	98.5 (97.7–99.2)
	48 h	0.668 (0.637–0.697)	0.089 (0.074–0.111)	93.8 (90.9–96.4)	19.4 (18.4–20.5)	5.6 (4.9–6.3)	98.4 (97.6–99.1)
	72 h	0.673 (0.641–0.705)	0.101 (0.082–0.128)	91.6 (88.4–94.6)	21.8 (20.7–22.9)	5.6 (4.9–6.3)	98.1 (97.3–98.8)
**Logistic regression**	0 h	0.630 (0.595–0.663)	0.096 (0.076–0.122)	74.4 (69.6–79.2)	40.3 (38.9–41.5)	6.0 (5.1–6.7)	96.9 (96.1–97.6)
	48 h	0.612 (0.580–0.646)	0.077 (0.064–0.099)	67.0 (61.6–72.4)	47.2 (45.8–48.5)	6.1 (5.3–6.9)	96.6 (95.8–97.2)
	72 h	0.620 (0.584–0.654)	0.083 (0.067–0.107)	71.8 (66.4–77.0)	40.5 (39.2–41.7)	5.8 (5.0–6.6)	96.6 (95.8–97.3)

External validation at MIMIC-IV (*n* = 5623; AKI prevalence 4.9%). Thresholds determined from the development set (LSTM-Attention: 0.817/0.801/0.724; Masked CNN: 0.622/0.548/0.532; ITE-Transformer: 0.789/0.694/0.677; XGBoost: 0.325/0.377/0.376; Logistic Regression: 0.495/0.555/0.506 for PH 0 h/48 h/72 h). Abbreviations as in Table 2.

### Online simulation performance

**Fig. 2:**
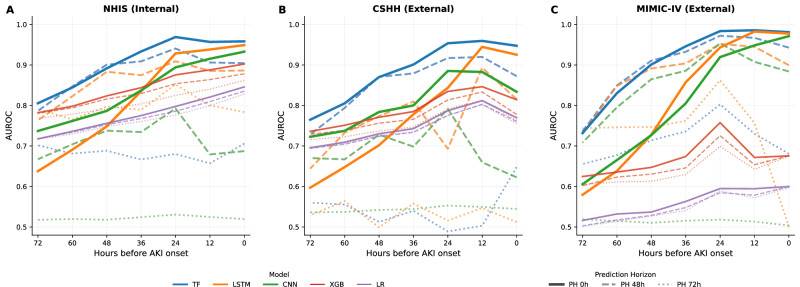
Online simulation performance across prediction horizons and sites. AUROC trajectories from 72 h before AKI onset to event time at NHIS (**A**), CSHH (**B**), and MIMIC-IV (**C**). Predictions were generated at 12 h intervals using matched reference times for non-AKI patients. Five model architectures are shown: ITE-Transformer (TF), LSTM-Attention (LSTM), Masked CNN (CNN), XGBoost (XGB), and Logistic Regression (LR). Line styles indicate prediction horizons: 0 h (solid), 48 h (dashed), and 72 h (dotted). Models trained at the 0 h horizon demonstrated consistent AUROC improvement toward onset across all sites, while models at longer horizons showed less stable trajectories.

Online simulation highlighted important differences in model behaviour that were not apparent from single-point evaluation alone (Fig. 2 and Supplementary Table S3). Models trained at 0 h prediction demonstrated clinical faithfulness—consistent improvement in AUROC as the time of AKI onset approached—across all three sites, which was statistically confirmed by Mann–Kendall trend testing (15/15 model–site combinations significant at PH = 0 h; median Kendall’s *τ* = 0.905, all *p* < 0.036; Supplementary Table S4).

At NHIS, the ITE-Transformer (0 h) showed an increase in AUROC from 0.806 at 72 h before AKI to 0.958 at the time of onset (ΔAUROC = 0.152). At CSHH, the same model demonstrated a similar trajectory (AUROC 0.765 to 0.947; ΔAUROC = 0.182), and at MIMIC-IV the pattern was most pronounced (AUROC 0.732 to 0.981; ΔAUROC = 0.249). This consistent cross-site improvement supports the robustness of clinical faithfulness as an evaluative concept. Baseline models also demonstrated significant trends at PH = 0 h but with smaller slopes (XGBoost median Sen’s slope 0.012 vs. ITE-Transformer 0.039 per 12-h interval), indicating less steep improvement trajectories.

In contrast, models trained at longer prediction horizons displayed less stable trajectories (PH = 48 h: 9/15 significant, median *τ* = 0.714; PH = 72 h: 7/15 significant, median *τ* = 0.429). At MIMIC-IV, the 72 h ITE-Transformer showed near-flat performance (AUROC 0.681–0.695) across all time points, while the LSTM-Attention (72 h) actually deteriorated from AUROC 0.738 at 72 h to 0.513 at the time of onset. The Masked CNN (72 h) performed near chance level across all sites (AUROC 0.503–0.532), indicating a fundamental inability to discriminate under continuous monitoring. At CSHH, 48 h models showed oscillating trajectories—for example, the LSTM-Attention (48 h) alternated between AUROC values of 0.498 and 0.851 across time points—suggesting unstable temporal behaviour that could contribute to inconsistent clinical alerts.

Notably, online simulation revealed a reversal of architecture rankings compared with single-point evaluation. Although Masked CNN achieved the highest single-point AUROC (0.961), it demonstrated the most severe performance degradation under continuous monitoring, particularly at 72 h prediction, where AUROC values approached chance level (0.503–0.546). Conversely, the ITE-Transformer, which ranked third in single-point discrimination among deep learning models (AUROC 0.924), exhibited the most stable and clinically faithful trajectories across all sites and prediction horizons. Baseline models showed intermediate behaviour: XGBoost exhibited universally significant increasing trends (*τ* = 0.714–1.000 across all PH–site combinations) but with consistently smaller magnitudes of improvement (ΔAUROC + 0.051 to + 0.120 at PH = 0 h vs. +0.153 to + 0.249 for ITE-Transformer). This ranking reversal underscores the importance of deployment-oriented evaluation in complementing conventional metrics for model selection.

Cross-site analysis further supported the generalisability of these observations. The magnitude of AUROC improvement from 72 h to onset for the ITE-Transformer (0 h) was consistent across populations: ΔAUROC of 0.152 at NHIS, 0.182 at CSHH, and 0.249 at MIMIC-IV. The larger improvement at MIMIC-IV likely reflects the ICU-only population, where more pronounced physiological derangements preceding AKI provide stronger signals for the model to detect. In contrast, the 72 h models showed no meaningful improvement at any site, confirming that the information available at this prediction horizon is insufficient for reliable continuous monitoring.

### Alert burden and deployment suitability

To assess deployment suitability, we evaluated alert burden using threshold sweep analysis across decision thresholds from 0.1 to 0.9 (Supplementary Table S5 and Supplementary Fig. S3). At a threshold of 0.5, the ITE-Transformer (0 h) achieved AKI detection rates of 72.4–99.6% with non-AKI alert rates of 7.1–7.6%, yielding NNE of 1.5–2.4 across sites. In contrast, Masked CNN was impractical for deployment, with non-AKI alert rates exceeding 98.6% and NNE of 17.6–564. Baseline models showed intermediate alert burden profiles (XGBoost NNE 4.1–173; logistic regression NNE 6.1–215). The threshold sweep analysis revealed that the ITE-Transformer maintained favourable sensitivity–specificity trade-offs across a wide range of thresholds, whereas Masked CNN and baseline models required substantially higher thresholds to achieve acceptable false positive rates.

### Calibration and clinical utility

**Fig. 3:**
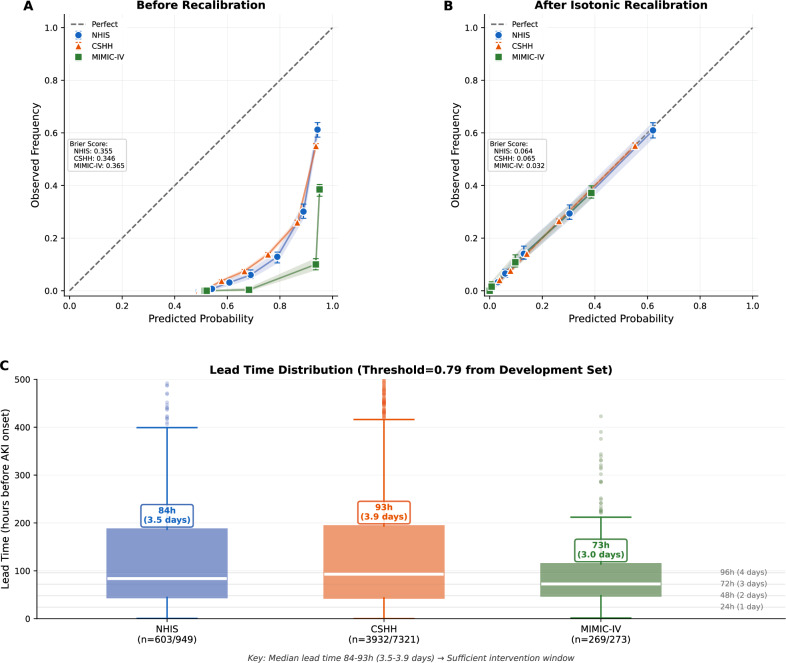
Clinical utility of the ITE-Transformer model (0 h prediction). **A** Calibration before recalibration, showing predicted probability versus observed AKI frequency across three sites. Points represent probability deciles with 95% confidence intervals. The diagonal dashed line indicates perfect calibration. **B** Calibration after isotonic regression recalibration (five-fold cross-validation per site), demonstrating substantially improved correspondence between predicted and observed frequencies. **C** Lead time distribution showing hours from first alert to AKI onset under continuous monitoring. Box plots display median, interquartile range, and 1.5 × IQR. AKI = acute kidney injury; CSHH = Chuncheon Sacred Heart Hospital; NHIS = National Health Insurance Service Ilsan Hospital.

Post-hoc isotonic regression recalibration substantially improved calibration without requiring model retraining (Fig. 3A, B, Supplementary Table S6, and Supplementary Fig. S4). For deep learning models, Brier scores were reduced by 76–94% across sites, while baseline models showed smaller improvements (49–88% for logistic regression; 32–89% for XGBoost). Using a probability threshold of 0.79, the ITE-Transformer (0 h) provided median lead times of 84 h at NHIS, 93 h at CSHH, and 73 h at MIMIC-IV (Fig. 3C)—suggesting that substantial advance warning may be achievable despite training at the 0 h prediction horizon.

## Discussion

In this multi-centre study, we developed and validated three deep learning and two baseline models for AKI prediction across three independent hospital settings and evaluated their performance under simulated continuous monitoring conditions. Our findings suggest that conventional single-point evaluation metrics may not fully capture a model’s suitability for deployment as a continuous monitoring tool. Although Masked CNN achieved the highest single-point discrimination (AUROC 0.961), the ITE-Transformer at 0 h prediction demonstrated more consistent and stable performance trajectories under simulated deployment conditions across all three validation sites, with the most favourable alert burden profile (NNE 1.5–2.4). The addition of baseline models confirmed that deep learning architectures provide substantial advantages in both discrimination and temporal stability, particularly at external sites.

A notable observation in our study was that models trained at longer prediction horizons (48 h and 72 h) exhibited less stable performance during online simulation, whereas models trained at 0 h prediction demonstrated clinical faithfulness. We believe this finding reflects the temporal dynamics of the AKI clinical process and the information available to each model during training. Models trained at 0 h prediction have access to the complete cascade of clinical deterioration preceding AKI diagnosis—including haemodynamic instability, nephrotoxic exposure, and inflammatory activation^17^. Crucially, this encompasses the full period of renal insult, which is estimated to begin approximately 48 h before the clinical diagnosis of AKI is established^17–19^. During continuous monitoring, these models can recognise these accumulating patterns whenever they appear, generating appropriately escalating risk scores as the underlying signal intensifies.

Models trained at 48 h prediction are positioned at approximately the onset of significant renal insult. These models can learn from early signals of renal injury, but may capture an incomplete picture of the clinical cascade. This partial exposure is consistent with their intermediate performance stability: at MIMIC-IV, the ITE-Transformer (48 h) achieved AUROC values ranging from 0.749 to 0.949, showing overall improvement but with more variability than the 0 h model. Models trained at 72 h prediction must identify risk from physiological data acquired before substantial renal dysfunction has developed. At this stage, the signal-to-noise ratio is inherently lower, and models may learn associations that do not reliably generalise across different patient populations or time points. This interpretation is supported by the near-random performance of several 72 h models during online simulation (AUROC 0.503–0.532 for Masked CNN across all sites), indicating that the patterns learned at this horizon do not translate to meaningful discrimination under continuous monitoring conditions.

Several prior studies have addressed continuous or sequential AKI prediction. Tomašev et al. demonstrated a recurrent neural network for AKI predictions up to 48 h in advance using data from the US Department of Veterans Affairs^7^, but did not systematically evaluate temporal performance trajectories or compare prediction horizons within a unified framework. Alfieri et al. developed a continuous AKI prediction model validated across 145 ICU centres in three countries, yet similarly relied on aggregate performance metrics without temporal trajectory analysis^5^. Our online simulation framework extends these works by providing a structured method for assessing temporal performance trajectories across multiple architectures and horizons simultaneously.

In the Korean context, Cho et al. developed a machine learning framework for AKI management across three Korean hospitals, reporting substantial performance drops during external validation^20^. Our study complements this by incorporating an international cohort (MIMIC-IV) and evaluating models under simulated deployment conditions. Koyner et al. developed one of the earlier machine learning models for inpatient AKI prediction using gradient boosting^21^, and recently demonstrated the value of multimodal deep learning incorporating unstructured clinical notes for AKI prediction, achieving earlier detection than creatinine-based criteria alone^22^. More broadly, systematic reviews have identified limited external validation and the absence of deployment-oriented evaluation as persistent gaps^12,13,23^.

The concept of clinical faithfulness—whereby model confidence increases appropriately as clinical deterioration progresses—has not, to our knowledge, been formally proposed as an evaluation criterion for continuous monitoring systems. Existing evaluation paradigms, including the recently updated TRIPOD + AI statement, focus primarily on discrimination and calibration at a single prediction point^24^. Our findings suggest that temporal performance stability may represent an additional dimension of model quality, particularly relevant for continuous monitoring deployment. A systematic comparison with prior studies is provided in Supplementary Table S7.

Several clinical implications emerge from our findings. First, online simulation represents a valuable addition to the evaluation toolkit for continuous monitoring systems. While single-point metrics remain important, they may not be sufficient on their own for informing deployment decisions. Our framework allows for the assessment of a model’s temporal stability, ensuring that automated alerts align with the progressive nature of the clinical process.

Second, our results suggest that shorter prediction horizons may, perhaps counterintuitively, provide more effective and reliable early warning. Models trained at 48 h or 72 h horizons attempt to capture faint, non-specific signals from the distant future and are more susceptible to noise, which may cause them to paradoxically misinterpret the strong signals of actual deterioration. In contrast, the 0 h model focuses on the clear cues of the AKI clinical process. Its sensitivity allows it to detect the subtle, accumulating precursors of AKI as they first emerge, achieving substantial lead times (73–93 h) by reliably recognising the early stages of an unfolding event rather than forecasting a speculative future.

Third, the clinical faithfulness of the 0 h model has significant implications for clinical adoption and the mitigation of alert fatigue. A model that exhibits a gradual, monotonic increase in risk scores is far more actionable for clinicians than one that produces erratic, sudden spikes^15,25^. A stable and predictable trajectory fosters greater clinical trust, as it mirrors the patient’s actual clinical course, allowing healthcare providers to intervene with confidence as the risk matures.

Fourth, the clinical faithfulness concept positions our framework within the detection paradigm rather than preventative care per se. While earlier detection may enable timely intervention, the translation of detection into improved outcomes requires prospective evaluation with defined action protocols (e.g., nephrology consultation triggers, fluid management algorithms, nephrotoxin avoidance bundles). Fifth, alert burden analysis revealed that model selection based solely on discrimination would lead to impractical deployment: Masked CNN, despite the highest AUROC, generated excessive false alerts (NNE 17.6–564), while the ITE-Transformer maintained clinically acceptable alert profiles (NNE 1.5–2.4). Sixth, isotonic regression recalibration appears to offer a practical approach for adapting models to different clinical settings without requiring complete model retraining^26^, with Brier score improvements of 76–94% for deep learning models observed in our study. Finally, the ranking reversal between single-point and online simulation evaluation has practical implications for how clinical informatics teams select and deploy models within EHR-integrated clinical decision support systems, suggesting that deployment decisions should incorporate temporal stability assessment alongside conventional discrimination metrics.

Several limitations of this study should be acknowledged. First, our findings are based on retrospective data, and prospective validation would be needed to confirm their clinical utility. Second, we did not compare our models against established clinical risk scores or novel biomarkers, which would be an important area for future investigation. Third, the 96 h minimum hospitalisation requirement for AKI cases may introduce selection bias toward persistent or hospital-acquired AKI events, excluding early-onset cases. This design choice was made to ensure sufficient pre-event data for online simulation and to exclude cases where renal insult may have preceded admission and thus would not be captured in the inpatient laboratory record. The resulting lead-time estimates should be interpreted within this context. Fourth, our models did not incorporate medication exposure or comorbidity data. Nephrotoxic drug exposure, in particular, is a well-established risk factor for AKI, and its omission may limit predictive performance. Future iterations should incorporate structured medication and comorbidity data where available. Fifth, the requirement of at least one measurement for each vital sign may introduce selection bias, as patients who are clinically stable may have fewer measurements. Additionally, the opportunistic nature of laboratory measurements in observational studies means that measurement frequency may itself be informative and correlated with disease severity. Sixth, the retrospective design does not allow us to determine whether the alerts generated by these models would influence clinical decision-making or improve patient outcomes. Seventh, our models adopt an “ignore treatment” strategy, learning from historical outcomes without modelling the effects of clinical interventions. Prediction-under-intervention modelling and prospective evaluation represent necessary future directions to determine how model-prompted behaviour changes affect both model performance and patient outcomes. Eighth, formal hyperparameter optimisation was not performed, and data splitting was conducted at the admission rather than patient level, which may have limited individual model performance. Our Korean cohorts may not fully capture global demographic diversity, though the inclusion of MIMIC-IV partially addresses this. Finally, although our study included data from three hospital systems in two countries, further validation across a broader range of clinical settings would strengthen the generalisability of these findings.

In conclusion, our study suggests that online simulation may serve as a valuable adjunct to conventional single-point evaluation for models intended for continuous monitoring deployment. The ITE-Transformer at 0 h prediction demonstrated more consistent deployment characteristics by aligning its predictive confidence with the temporal dynamics of the AKI clinical process, while also achieving the most favourable alert burden profile among all architectures evaluated. These findings support the consideration of deployment-oriented evaluation—incorporating temporal trajectory analysis, alert burden assessment, and formal trend testing—alongside static accuracy metrics when assessing clinical prediction models, and suggest that shorter prediction horizons may warrant further investigation for continuous AKI monitoring systems.

## Methods

### Study design and data sources

**Fig. 4:**
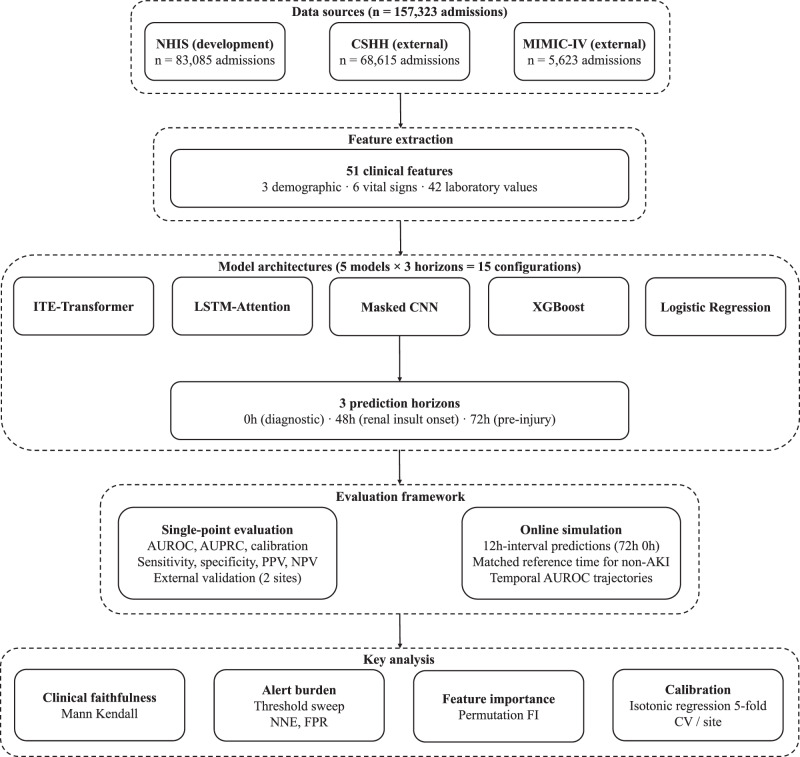
Study design and data processing pipeline. Overview of the study pipeline from raw EHR data through feature extraction (51 clinical features), architecture-specific preprocessing, model training (five models × three prediction horizons = 15 configurations), and the dual evaluation framework (single-point evaluation and online simulation).

This retrospective cohort study used electronic health record data from three independent cohorts (Fig. 4): National Health Insurance Service Ilsan Hospital (NHIS; Goyang-si, Korea) for model development, and Chuncheon Sacred Heart Hospital (CSHH; Chuncheon-si, Korea) and Medical Information Mart for Intensive Care IV (MIMIC-IV; Boston, USA)^27^ for external validation. NHIS and CSHH included both general ward and intensive care unit settings; MIMIC-IV comprised intensive care unit data only. The study was approved by the institutional review boards at NHIS (IRB 2022-11-032) and CSHH (IRB 2023-08-017).

### Study population

We included adult patients (aged 19 years or older) with hospital stays of at least 24 h for non-AKI cases and at least 96 h for AKI cases. Patients were required to have at least one measurement for each vital sign (systolic and diastolic blood pressure, pulse rate, respiratory rate, and body temperature). The patient selection process is detailed in Supplementary Fig. S1.

### Outcome definition

AKI was defined according to the Kidney Disease: Improving Global Outcomes (KDIGO) criteria, incorporating both serum creatinine and urine output parameters^28,29^. Baseline creatinine was determined using a rolling 48 h minimum window approach, defined as the minimum serum creatinine value observed within the preceding 48 h at each evaluation time point. This approach differs from the acute dialysis quality initiative (ADQI) consensus recommendation of a 7-day window and was adopted to accommodate data availability heterogeneity across cohorts while maintaining clinical relevance. The AKI outcome definition remained identical across all prediction horizons; the horizons differed only in the amount of clinical data available at the time of prediction. We developed models at three prediction horizons: diagnostic (0 h), 48 h, and 72 h. All models were designed for continuous real-time risk assessment throughout hospitalisation.

### Input features and preprocessing

Models utilised 51 clinical features comprising 3 demographic variables (age, sex, and body-mass index), 6 vital signs, and 42 laboratory values (Supplementary Table S8). We adopted an inclusive feature selection strategy, incorporating all routinely available clinical variables from the electronic health records at our study sites. This approach was chosen to maximise the information available to each model and to avoid premature exclusion of potentially informative features. Urine output was not included among the input features because it is not systematically measured outside of intensive care settings, making it unreliable as a predictor variable in general ward populations. Three variables (total cholesterol, platelet distribution width, procalcitonin) were not routinely collected in MIMIC-IV. Missingness rates during hospitalisation and measurement frequencies for all features are reported in Supplementary Table S9. Time-series data were processed according to the requirements of each architecture: hourly resampling was applied for the CNN model, while the LSTM-Attention and ITE-Transformer utilised raw irregular sequences through initial triplet embedding^30^. Missing data were handled differently across architectures. The ITE-Transformer and LSTM-Attention inherently accommodate missingness by operating only on observed values within the triplet embedding framework, where unobserved time points are excluded from the input sequence^30^. The Masked CNN, by contrast, received zero-filled time series concatenated with binary masking vectors indicating whether each variable was observed or missing at each time step, allowing the network to learn the informative missingness patterns during training^31^.

### Model development

Three deep learning architectures were developed (Supplementary Fig. S5): (1) LSTM-Attention: a three-layer long short-term memory network with an attention mechanism, utilising initial triplet embedding for irregular time-series input; (2) Masked CNN: a masked residual one-dimensional convolutional network designed to handle irregular time-series data through dynamic masking^32^; and (3) ITE-Transformer: an initial triplet embedding transformer that processes clinical data as event triplets (feature type, timestamp, and value) without requiring resampling^33^. These architectures represent the spectrum of approaches for handling variable-length clinical time-series data^34^.

Two baseline models were additionally trained to enable comparison with non-deep-learning approaches: XGBoost (gradient boosting) and logistic regression, using the same feature set and prediction horizons.

Each architecture was trained at three prediction horizons, yielding fifteen model configurations (five models × three prediction horizons). Models were trained using PyTorch with the AdamW optimiser (learning rate 0.0001), a batch size of 32, and a combined loss of binary cross-entropy and cosine similarity, for up to 50 epochs with early stopping (patience of 10 epochs on validation loss). These hyperparameters were selected based on established defaults in the clinical time-series literature^30,34^ and preliminary experiments on the NHIS validation set. Formal hyperparameter optimisation (e.g., grid search, Bayesian optimisation) was not performed, which we acknowledge as a limitation. A fixed random seed (1004) was used across all experiments. The NHIS dataset was partitioned in an 8:1:1 ratio for training, validation, and testing at the admission level to prevent data leakage. While patient-level splitting would provide stricter leakage prevention, each admission represents a clinically independent episode with distinct temporal patterns, and the proportion of patients with multiple admissions was sufficiently low to minimise leakage risk. Detailed descriptions of data preprocessing, model architectures, and training procedures are provided in the Supplementary Note.

### Online simulation framework

To evaluate performance under conditions that approximate clinical deployment, we developed an online simulation framework designed to replicate the operation of a continuous early warning system. For patients who developed AKI, predictions were generated at 12 h intervals from 72 h before onset to the time of the event, yielding seven evaluation points per patient (72 h, 60 h, 48 h, 36 h, 24 h, 12 h, and 0 h). For patients who did not develop AKI, reference times were selected using matched temporal ratios based on the hospitalisation onset-to-event distribution of AKI patients, and predictions were generated at corresponding 12 h intervals. A sensitivity analysis comparing matched reference times with discharge-fixed reference times confirmed that trajectory patterns were robust across both methods (Supplementary Fig. S6). At each evaluation point, models received all clinical data available up to that moment. AUROC was calculated at each time point using predictions from both AKI and non-AKI patients, generating temporal performance trajectories.

Clinical faithfulness was operationalised as a statistically significant monotonic increasing trend in AUROC toward the time of AKI onset, assessed using the Mann–Kendall trend test (Kendall’s *τ*) with Sen’s slope to quantify the rate of improvement per 12 h interval (Supplementary Table S4). Models demonstrating clinical faithfulness were considered potentially more suitable for deployment, as this behaviour would be expected to provide increasingly reliable alerts at the time when clinical intervention may be most beneficial. The simulation was conducted separately for each site.

### Statistical analysis

Bootstrap resampling (*n* = 1000) was used to generate 95% confidence intervals. Calibration was assessed using Brier scores before and after isotonic regression recalibration. Isotonic regression was fitted using five-fold cross-validation on each site’s data separately to prevent information leakage between calibration fitting and evaluation (Supplementary Table S6 and Supplementary Fig. S4). Lead time was calculated as the interval between the first positive alert and the onset of AKI. Permutation feature importance (PFI) was computed by measuring the AUROC drop upon patient-level permutation of each feature, repeated five times to estimate variability (Supplementary Fig. S7). Model performance under reduced feature sets was assessed using zero-masking of features outside the top-K% ranked by PFI (Supplementary Table S10). Alert burden was quantified using the number needed to evaluate (NNE = 1/PPV), and threshold sweep analysis was conducted across decision thresholds from 0.1 to 0.9, evaluating sensitivity, false positive rate, and NNE for all models (Supplementary Table S5 and Supplementary Fig. S3). Analyses were conducted using Python 3.13.7 with PyTorch 2.8.0 and scikit-learn 1.7.0.

## Supplementary information

Supplementary materials

## Data Availability

MIMIC-IV data are publicly available via PhysioNet (https://physionet.org/). NHIS Ilsan Hospital and Chuncheon Sacred Heart Hospital data are not publicly available due to institutional data use agreements and patient privacy regulations, but are available from the corresponding author on reasonable request with appropriate IRB approval.

## Code availability

The code used in this study is publicly available at https://github.com/Jovinus/aki-timeseries-continuous-monitoring.
