# Supplementary material for: Deep learning models for acute kidney injury prediction: multi-center external validation and evaluation under simulated continuous monitoring conditions
Source: NPJ Digit Med. 2026 May 8;9:544. doi: 10.1038/s41746-026-02722-2 (PMC13369875; doi:10.1038/s41746-026-02722-2)
Supplement: Supplementary file 1 — Supplementary materials [file 41746_2026_2722_MOESM1_ESM.pdf]

SUPPLEMENTARY MATERIALS

Deep Learning Models for Acute Kidney Injury Prediction: Multi-Center External Validation and Evaluation Under Simulated Continuous Monitoring Conditions

TABLE OF CONTENTS

Supplementary Note

– Detailed Study Design and Data Sources
– Data Preprocessing and Feature Engineering
– Model Architecture Details
– Training Procedures
– Online Simulation Protocol

Supplementary Figures

– Figure S1. Patient Selection Flow Chart
– Figure S2. External Validation Discrimination by Site
– Figure S3. Threshold sweep analysis (PH=0h, at onset)
– Figure S4. Calibration Before and After Recalibration
– Figure S5. Model Architecture Diagrams
– Figure S6. Reference time sensitivity analysis (PH=0h)
– Figure S7. Permutation feature importance for the ITE-Transformer

Supplementary Tables

– Table S1. Detailed Baseline Characteristics
– Table S2. Single-Point Discrimination Performance of All Models
– Table S3. Online Simulation AUROC Trajectories (PH=0h, Matched Reference)
– Table S4. Mann–Kendall Trend Test for Clinical Faithfulness (AUROC)
– Table S5. Alert Burden Analysis (PH=0h, Threshold=0.5)
– Table S6. Calibration Metrics (Brier Score) Before and After Isotonic Recalibration
– Table S7. Comparison with Prior AKI Prediction Studies
– Table S8. Complete List of Input Features
– Table S9. Missingness Rates During Hospitalisation and Measurement Frequency by Feature and Cohort
– Table S10. Model Performance with Reduced Feature Sets (PH=0h)

SUPPLEMENTARY NOTE

1. Detailed Study Design and Data Sources

1.1 Study Period and Settings

This retrospective cohort study was conducted from November 2022 to November 2025. Patient data were collected from electronic health records spanning the period from 2015 to 2021. Three independent hospital sites were included:

National Health Insurance Service Ilsan Hospital (NHIS)

- Location: Goyang-si, Gyeonggi-do, Republic of Korea
- Setting: General ward and intensive care unit
- Role: Development cohort (model training and internal validation)
- Institutional review board approval: 2022-11-032

Chuncheon Sacred Heart Hospital (CSHH)

- Location: Chuncheon-si, Gangwon-do, Republic of Korea
- Setting: General ward and intensive care unit
- Role: External validation cohort
- Institutional review board approval: 2023-08-017

Medical Information Mart for Intensive Care IV (MIMIC-IV)

- Location: Beth Israel Deaconess Medical Center, Boston, MA, United States
- Setting: Intensive care unit only
- Role: External validation cohort
- Access: PhysioNet credentialed user agreement

1.2 Patient Cohort Construction

Patient cohorts were identified from electronic health records using admission, discharge, and location transfer data. We adopted a unified approach to cohort construction that captured the complete patient trajectory, including ward transfers, rather than creating separate intensive care unit and general ward cohorts. This approach was intended to allow models to learn patterns across different care settings, which may better reflect the clinical reality of patient movement within hospitals.

Initial screening:

- NHIS: 143,520 visits
- CSHH: 90,569 visits
- MIMIC-IV: 60,251 visits

Exclusions based on length-of-stay criteria:

- NHIS: 59,772 visits excluded
- CSHH: 17,838 visits excluded
- MIMIC-IV: 54,588 visits excluded

Exclusions for insufficient vital sign recordings:

- NHIS: 633 visits excluded
- CSHH: 4,116 visits excluded
- MIMIC-IV: 40 visits excluded

Final cohorts:

- NHIS: 83,085 visits
- CSHH: 68,615 visits
- MIMIC-IV: 5,623 visits

2. Data Preprocessing and Feature Engineering

2.1 Temporal Feature Processing

Temporal feature engineering was tailored to each model architecture in order to optimise performance while accommodating the different approaches to handling irregular time-series data that are characteristic of clinical settings.

For Masked CNN model:

- – Historical data window: up to 256 hours (approximately 10.7 days) before each prediction time point
- – Resampling: hourly intervals
- – Value selection: last observed value within each hourly interval
- – This approach provides the regularly sampled input required by this architecture

For LSTM-Attention and ITE-Transformer architectures:

- – No resampling or temporal truncation applied
- – All available historical data utilised directly
- – Native handling of irregularly sampled data through event-based representation

2.2 Normalisation

All continuous features were normalised using robust scaling:

- – Central tendency: median (rather than mean)
- – Spread: interquartile range (rather than standard deviation)

Normalisation parameters were computed exclusively from the training dataset and subsequently applied to the validation and external test datasets to prevent data leakage.

2.3 Clinical Outlier Handling

Clinically implausible values identified as likely measurement errors were reviewed by physicians and removed prior to analysis. These included:

- – Physiologically implausible values (e.g., negative vital signs)
- – Probable data entry errors (e.g., decimal point misplacements)
- – Equipment malfunction artefacts

2.4 Missing Data Handling

Missing data were handled differently across architectures, with no explicit imputation performed for any model:

ITE-Transformer and LSTM-Attention:

- – These architectures inherently accommodate missingness by operating only on observed values within the triplet embedding framework, where unobserved time points are excluded from the input sequence

Masked CNN:

- – Received zero-filled time series concatenated with binary masking vectors indicating whether each variable was observed or missing at each time step, allowing the network to learn informative missingness patterns during training

XGBoost and logistic regression:

- – Used the last observed value for each feature at the time of prediction. Features with no prior observation were set to median

3. Model Architecture Details

3.1 Masked CNN Architecture

The Masked CNN architecture was developed to accommodate the challenges of irregularly sampled clinical time-series data, employing deep residual learning for temporal feature extraction.

Input processing:

– Two input types: static demographic features and dynamic time-series features
– Time-series structured as one-dimensional signal sequences
– Dynamic masking mechanism for variable-length hospital episodes
– Binary masks generated based on valid (non-padded) time steps

Core network architecture:

– Initial convolutional block: kernel size 5, stride 1
– Max-pooling layer following initial convolution
– Four stages of stacked residual blocks
– Channel dimensions: 64 → 128 → 192 → 256

Residual block design (bottleneck-like for time series):

– 1D convolution with large receptive field (kernel size 13)
– Root mean square normalisation (RMSNorm)
– Gaussian error linear unit (GELU) activation
– Residual connections with 1×1 convolutions for channel alignment

Output processing:

– Masked global average pooling (averaging over valid time steps only)
– Temporal embedding concatenated with demographic features
– Multi-layer perceptron (MLP) classification head
– Components: linear layers, GELU activations, dropout (p=0.1)

3.2 ITE-Transformer Architecture

The ITE-Transformer was designed to process sparse, irregularly sampled multivariate clinical events without requiring imputation, representing patient data as sequences of clinical events.

Event representation (triplet format):

– Feature type: categorical identifier for the clinical variable
– Timestamp: time of measurement
– Measured value: the recorded clinical value

Embedding scheme (sum of three representations):

– Type embedding: categorical embedding vector
– Time embedding: continuous embedding via MLP (Linear→Tanh→Linear)
– Value embedding: continuous embedding via MLP (Linear→Tanh→Linear)

Sequence processing:

– Learnable [CLS] token prepended to event sequence
– Standard transformer encoder stack with multi-head self-attention
– GELU activations in feed-forward networks
– Padding masks applied to handle varying sequence lengths

Classification:

– Final hidden state of [CLS] token used as aggregate representation
– Classification head: Linear→Tanh→Dropout→Linear
– Output: AKI risk probability

3.3 LSTM-Attention Architecture

Architecture components:

- – Input representation: initial triplet embedding shared with ITE-Transformer (feature type, timestamp, and value embeddings)
- – Three stacked LSTM layers
- – Hidden state dimension: equal to model embedding size
- – Sequential processing of multivariate time-series inputs
- – Attention mechanism for temporal feature weighting
- – Final hidden state passed through a projection head for classification

4. Training Procedures

Models were trained using the following configuration:

- – Framework: PyTorch 2.8.0
- – Optimiser: AdamW with learning rate 0.0001
- – Batch size: 32
- – Training epochs: 50
- – Loss function: combined binary cross-entropy and cosine similarity
- – Early stopping: patience of 10 epochs on validation loss
- – Random seed: 1004 (fixed across all experiments)

SUPPLEMENTARY FIGURES

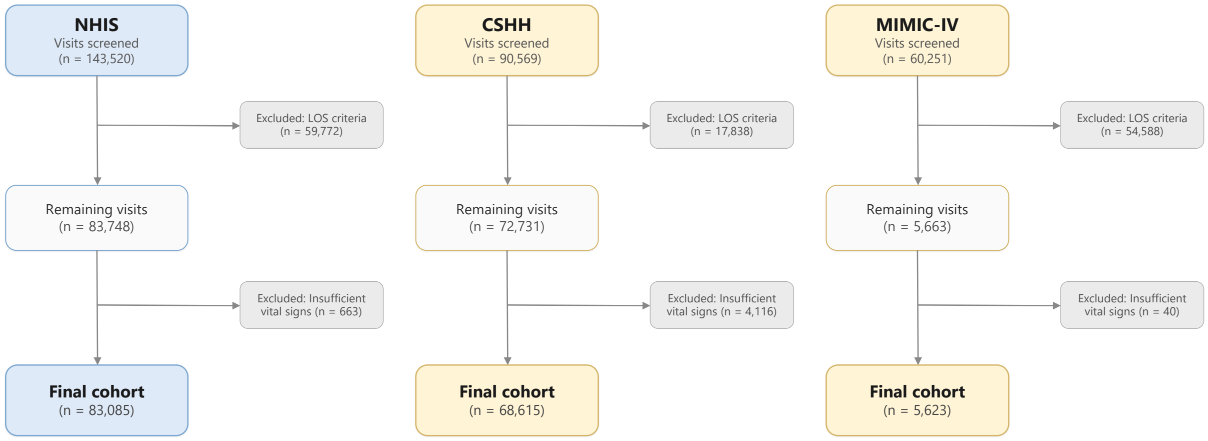

Supplementary Figure S1. Patient Selection Flow Chart

Flow diagram illustrating the patient selection process across three independent cohorts: NHIS (National Health Insurance Service, South Korea), CSHH (Chuncheon Sacred Heart Hospital, Hallym University, South Korea), and MIMIC-IV (Medical Information Mart for Intensive Care IV, United States). Initially, 143,520 visits at NHIS, 90,569 visits at CSHH, and 60,251 visits at MIMIC-IV were screened. Patients were excluded based on length-of-stay criteria (NHIS: n=59,772; CSHH: n=17,838; MIMIC-IV: n=54,588) and insufficient vital sign recordings (NHIS: n=633; CSHH: n=4,116; MIMIC-IV: n=40). The final analysis included 83,085 visits from NHIS, 68,615 visits from CSHH, and 5,623 visits from MIMIC-IV, totalling 157,323 visits.

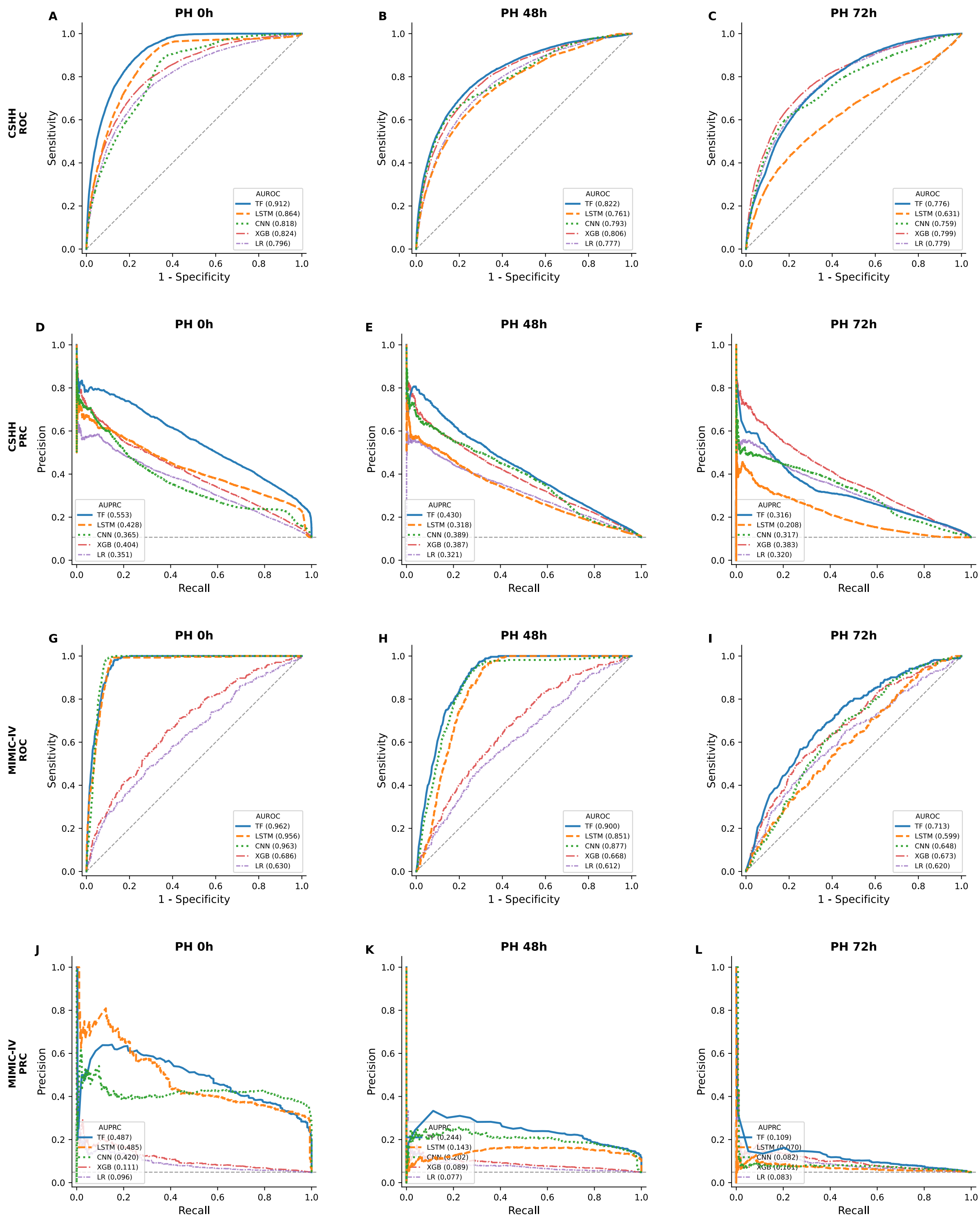

Supplementary Figure S2. External Validation Discrimination by Site

External validation discrimination across prediction horizons and sites. Receiver operating characteristic (ROC) curves and precision–recall curves (PRC) for all five models at CSHH (A–F) and MIMIC-IV (G–L). Columns represent prediction horizons: 0-hour (A, D, G, J), 48-hour (B, E, H, K), and 72-hour (C, F, I, L). Five model architectures are shown: ITE-Transformer (TF; blue solid), LSTM-Attention (LSTM; orange dashed), Masked CNN (CNN; green dotted), XGBoost (XGB; red dash-dot), and Logistic Regression (LR; grey dash-dot-dot). AUROC and AUPRC values are reported in each panel legend. At MIMIC-IV, deep learning models substantially outperformed baseline models at the 0-hour horizon (AUROC 0.956–0.963 vs. 0.630–0.686), while the performance gap narrowed at longer horizons.

(A) NHIS (internal)

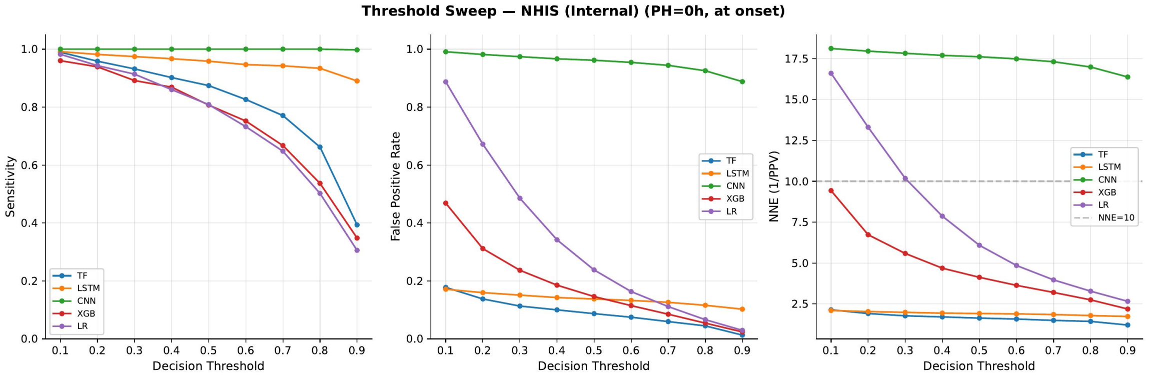

(B) CSHH (external)

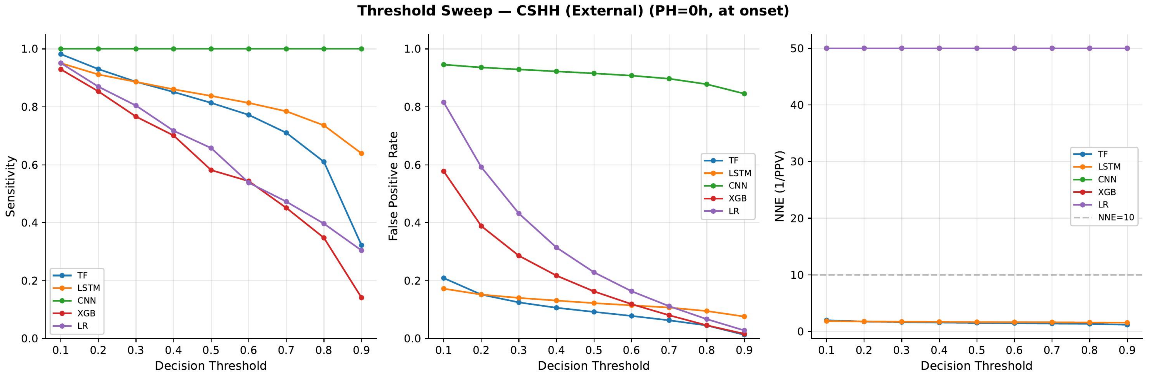

(C) MIMIC-IV (external)

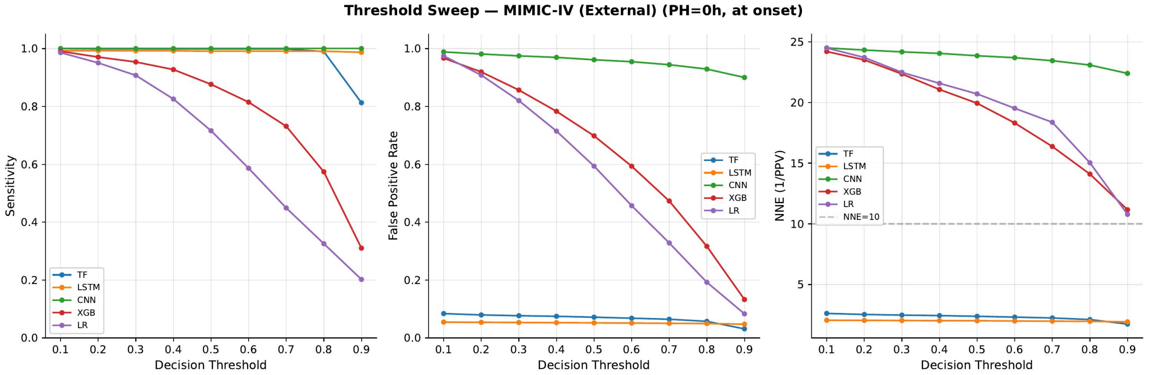

Supplementary Figure S3. **Threshold sweep analysis (PH=0h, at onset)**

Sensitivity (left), false positive rate (centre), and number needed to evaluate (NNE; right) as a function of decision threshold (0.1–0.9) for all five models. Dashed grey line in NNE panels indicates NNE = 10. (A) NHIS. (B) CSHH. (C) MIMIC-IV.

(A) Prediction Horizon = 0h

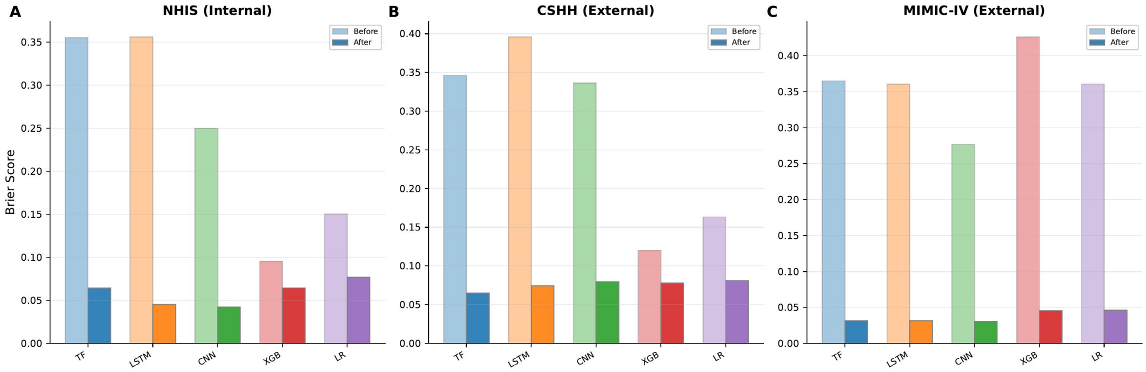

(B) Prediction Horizon = 48h

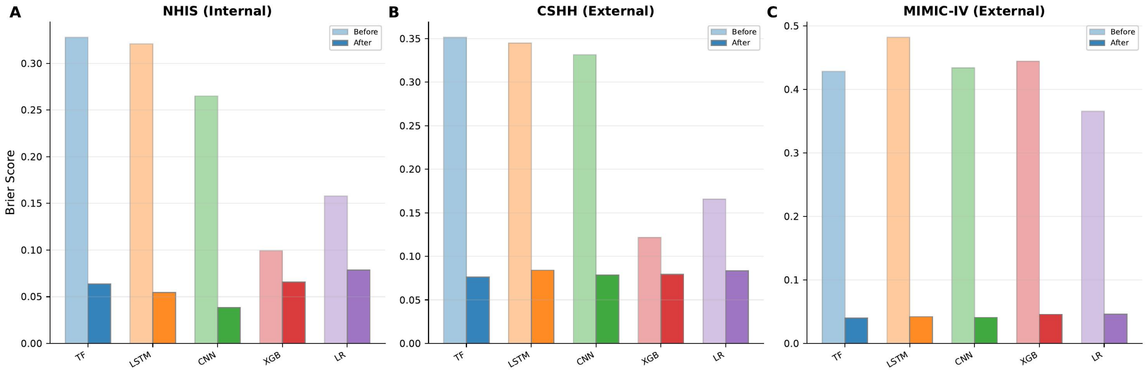

(C) Prediction Horizon = 72h

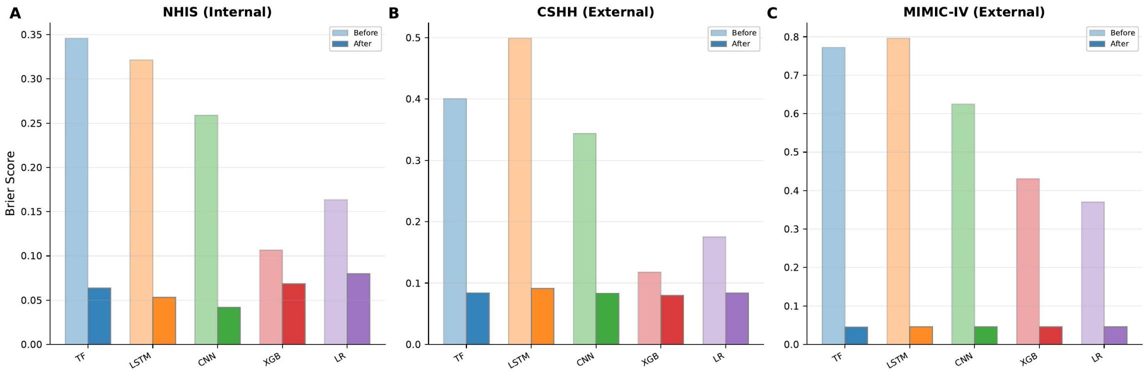

Supplementary Figure S4. Calibration Before and After Recalibration

Brier scores before (light bars) and after (dark bars) isotonic regression recalibration for all five models across NHIS (left), CSHH (centre), and MIMIC-IV (right). Recalibration was performed using five-fold cross-validation per site. (A) Prediction horizon = 0h. (B) Prediction horizon = 48h. (C) Prediction horizon = 72h.

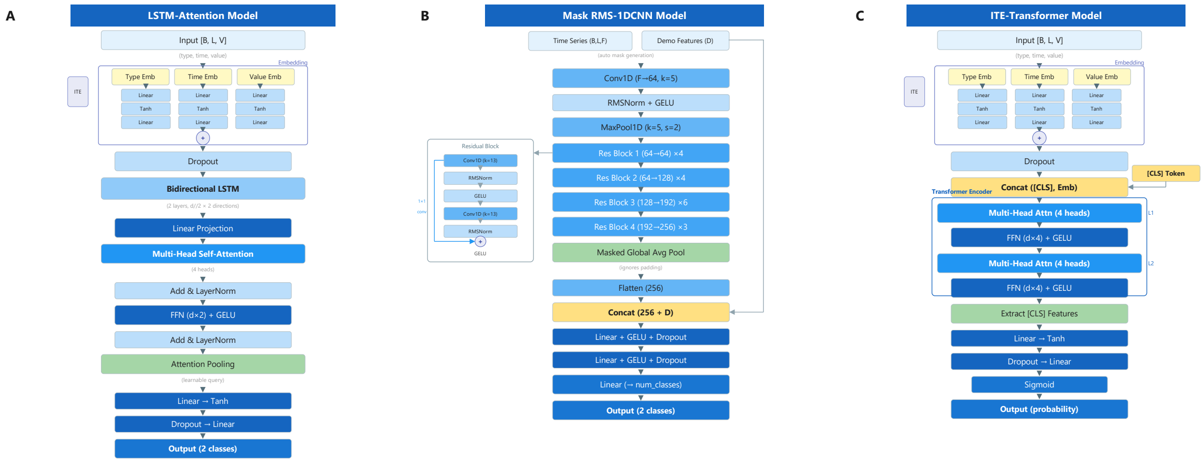

Supplementary Figure S5. Model Architecture Diagrams

Schematic diagrams of the three deep learning architectures: (A) LSTM-Attention, (B) Masked-CNN, and (C) ITE-Transformer.

(A) NHIS (internal)

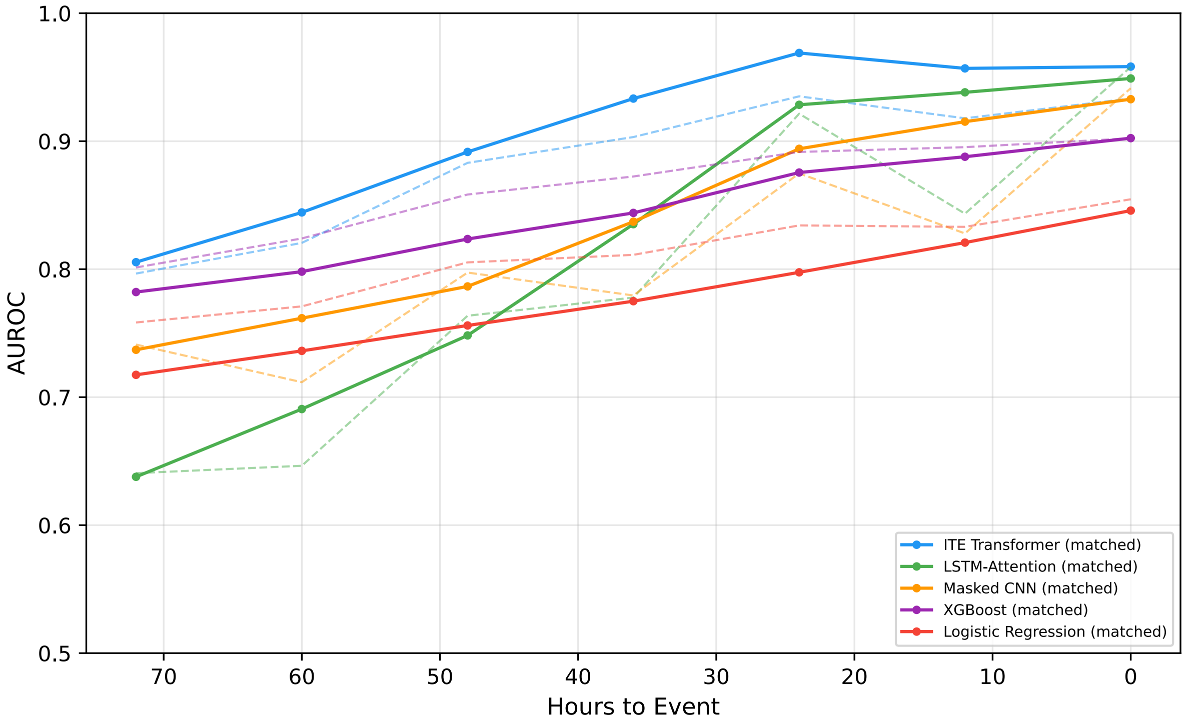

(B) CSHH (external)

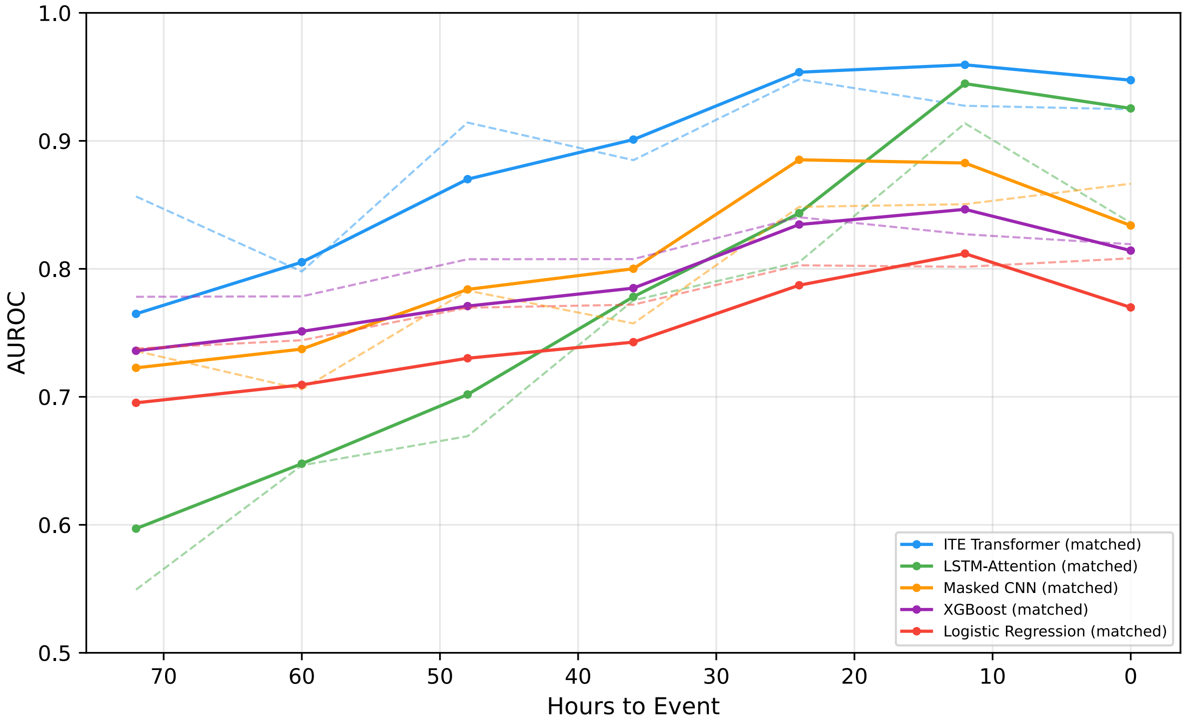

(C) MIMIC-IV (external)

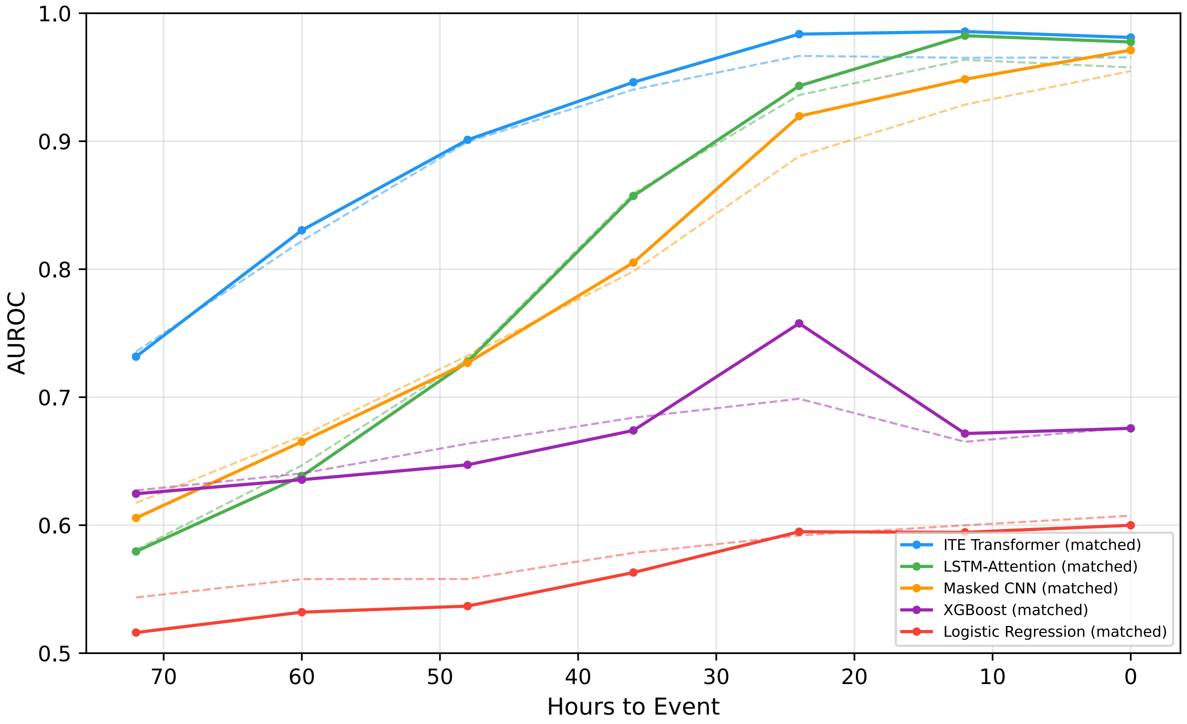

Supplementary Figure S6. Reference time sensitivity analysis (PH=0h)

Online simulation AUROC trajectories comparing matched reference time (solid lines) and discharge-fixed reference time (dashed lines). Similar trajectory patterns across both methods confirm robustness. (A) NHIS. (B) CSHH. (C) MIMIC-IV.

(A) PH = 0h, NHIS (internal)

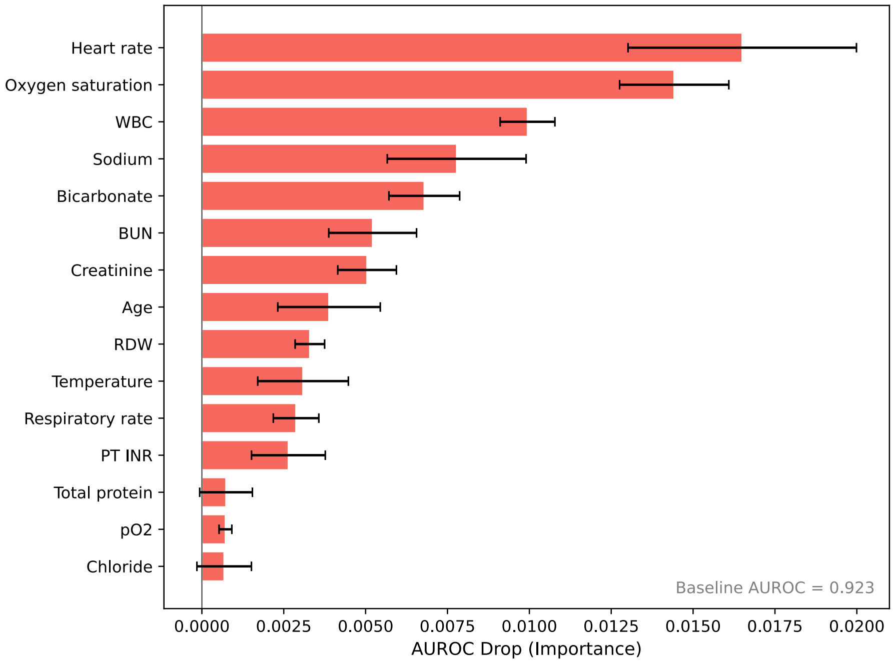

(B) PH = 0h, CSHH (external)

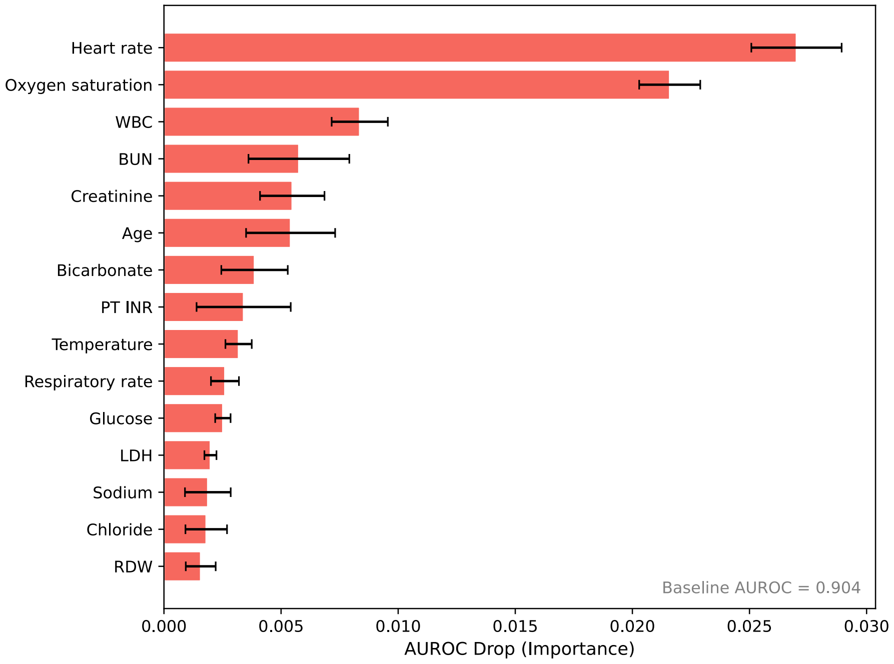

(C) PH = 0h, MIMIC-IV (external)

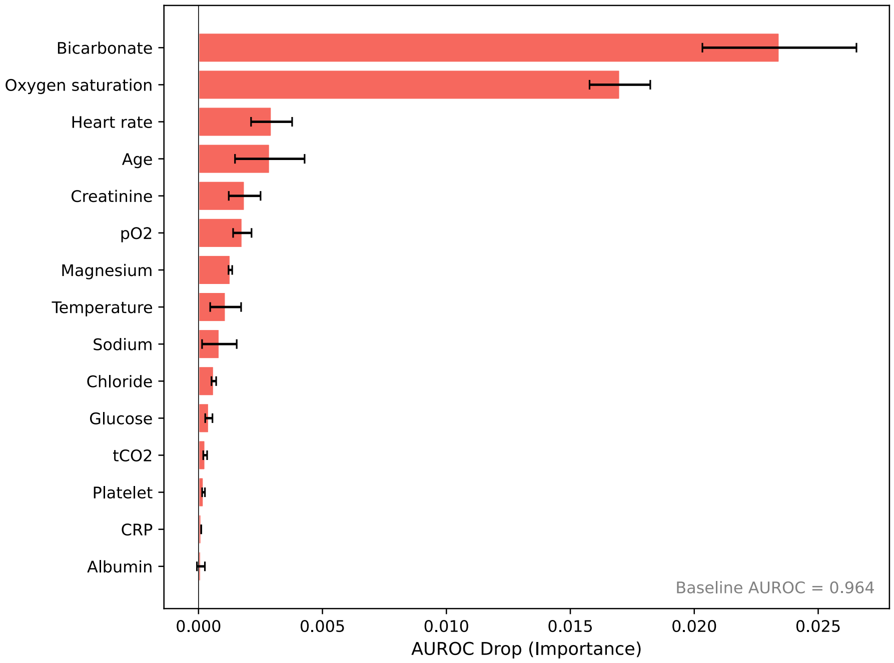

(D) PH = 48h, NHIS (internal)

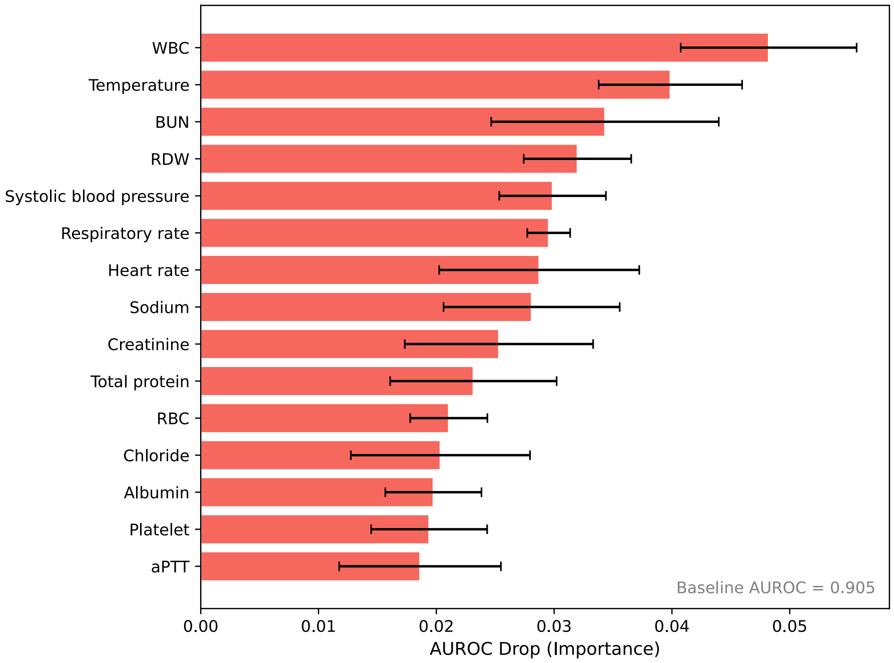

(E) PH = 72h, NHIS (internal)

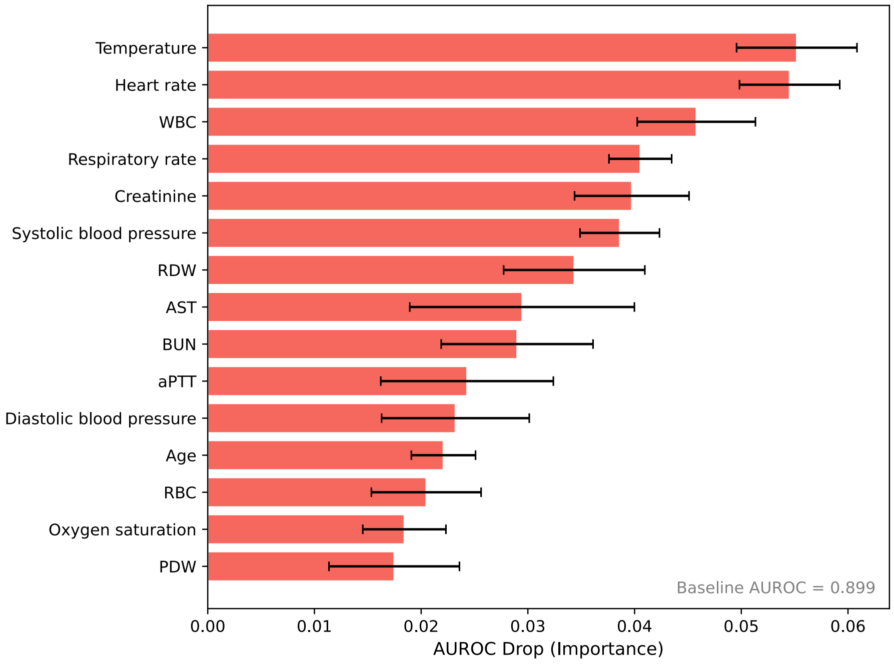

Supplementary Figure S7. Permutation feature importance for the ITE-Transformer

Top 15 features ranked by AUROC drop upon patient-level permutation. Error bars represent standard deviation across 5 repeats. Baseline AUROC is shown in the panel title. (A) PH = 0h, NHIS. (B) PH = 0h, CSHH. (C) PH = 0h, MIMIC-IV. (D) PH = 48h, NHIS. (E) PH = 72h, NHIS.

SUPPLEMENTARY TABLES

Supplementary Table S1. Detailed Baseline Characteristics

		Overall	NHIS (n=83085)	CSHH (n=68615)	MIMIC-IV (n=5623)	p-value
Age (years)		58.5 (18.4)	59.7 (17.4)	56.9 (19.7)	60.3 (16.4)	<0.001
Sex (n)	**Male**	78637 (50.0)	38875 (46.8)	36436 (53.1)	3326 (59.1)	<0.001
	**Female**	78686 (50.0)	44210 (53.2)	32179 (46.9)	2297 (40.9)	
BMI (kg/m²)		23.8 (4.0)	23.7 (3.8)	23.6 (3.9)	26.6 (6.6)	<0.001
Length of stay (days)		4.0 [2.0,8.0]	4.6 [2.8,9.0]	4.0 [2.0,7.0]	1.6 [1.2,2.5]	<0.001
Systolic blood pressure (mmHg)		127.7 (21.0)	131.8 (20.7)	123.2 (20.1)	123.5 (24.8)	<0.001
Diastolic blood pressure (mmHg)		76.0 (12.8)	77.6 (13.0)	74.7 (11.6)	69.4 (17.9)	<0.001
Heart rate (bpm)		82.7 (16.0)	82.7 (15.9)	82.3 (15.8)	88.4 (20.0)	<0.001
Respiratory rate (/min)		19.5 (2.4)	19.0 (2.1)	20.2 (2.0)	19.1 (5.9)	<0.001
Oxygen saturation (%)		96.8 (2.9)	96.6 (3.1)	96.8 (2.7)	97.3 (3.4)	<0.001
Temperature (°C)		36.8 (0.6)	36.8 (0.5)	36.8 (0.6)	36.7 (0.8)	<0.001
WBC (×10³/μL)		7.4 [5.6,10.1]	7.7 [5.8,10.4]	7.0 [5.3,9.5]	9.9 [7.1,13.7]	<0.001
Haemoglobin (g/dL)		12.7 [11.1,14.0]	12.5 [11.1,13.9]	13.0 [11.5,14.3]	10.1 [8.5,11.8]	<0.001
Haematocrit (%)		37.7 [33.5,41.4]	37.3 [33.3,40.9]	38.4 [34.4,42.1]	31.0 [26.5,35.8]	<0.001
RBC (×10⁶/μL)		4.1 [3.6,4.6]	4.1 [3.6,4.5]	4.2 [3.8,4.6]	3.4 [2.9,3.9]	<0.001
RDW (%)		13.1 [12.5,14.2]	13.1 [12.5,14.1]	13.0 [12.4,14.1]	14.7 [13.4,16.5]	<0.001
Platelet (×10³/μL)		225.0 [179.0,275.0]	224.0 [180.0,273.0]	228.0 [182.0,279.0]	193.0 [137.0,265.0]	<0.001
PDW (%)		10.7 [9.7,12.0]	10.8 [9.8,12.0]	10.5 [9.4,11.8]	Not available	
PT INR		1.1 [1.0,1.1]	1.0 [1.0,1.1]	1.1 [1.0,1.1]	1.3 [1.1,1.5]	<0.001
aPTT (sec)		28.9 [26.3,32.1]	27.6 [25.3,30.6]	30.3 [27.8,33.0]	30.8 [27.3,36.0]	<0.001
BUN (mg/dL)		14.7 [11.2,19.8]	14.6 [11.0,19.8]	14.7 [11.4,19.3]	19.0 [12.0,35.0]	<0.001
Creatinine (mg/dL)		0.8 [0.7,1.0]	0.8 [0.6,1.0]	0.9 [0.7,1.0]	0.9 [0.7,1.5]	<0.001
AST (U/L)		24.0 [19.0,34.0]	25.0 [20.0,35.0]	24.0 [19.0,33.0]	30.0 [20.0,56.0]	<0.001
ALT (U/L)		19.0 [13.0,30.0]	19.0 [14.0,29.0]	19.0 [13.0,29.0]	22.0 [14.0,42.0]	<0.001
GGT (U/L)		28.0 [17.0,61.0]	33.0 [17.0,107.0]	28.0 [17.0,55.0]	62.0 [23.5,172.0]	<0.001
Alkaline phosphatase (U/L)		76.0 [61.0,100.0]	74.0 [58.0,103.0]	77.0 [62.0,98.0]	90.0 [65.0,133.0]	<0.001
Total bilirubin (mg/dL)		0.6 [0.5,0.9]	0.7 [0.5,1.0]	0.6 [0.4,0.8]	0.5 [0.3,0.9]	<0.001
Glucose (mg/dL)		119.0 [102.0,151.0]	122.0 [104.0,154.0]	117.0 [100.0,148.0]	122.0 [100.0,155.0]	<0.001
Total protein (g/dL)		6.8 [6.3,7.2]	6.7 [6.2,7.2]	6.8 [6.4,7.2]	6.0 [5.4,6.6]	<0.001
Albumin (g/dL)		4.0 [3.5,4.3]	3.8 [3.3,4.1]	4.1 [3.8,4.4]	3.1 [2.6,3.6]	<0.001
Uric acid (mg/dL)		4.7 [3.7,6.0]	4.5 [3.5,5.8]	4.8 [3.8,6.0]	5.0 [2.9,6.9]	<0.001
Calcium (mg/dL)		8.9 [8.4,9.3]	8.6 [8.2,9.0]	9.0 [8.6,9.4]	8.5 [8.0,9.0]	<0.001
Phosphate (mg/dL)		3.4 [2.9,3.9]	3.5 [3.0,4.0]	3.4 [2.9,3.9]	3.5 [2.9,4.3]	<0.001

Sodium (mg/dL)		139.0 [137.0,141.0]	138.0 [136.0,140.0]	141.0 [138.0,143.0]	138.0 [135.0,140.0]	<0.001
Potassium (mg/dL)		4.1 [3.8,4.4]	4.0 [3.8,4.3]	4.1 [3.8,4.4]	4.2 [3.8,4.6]	<0.001
Chloride (mg/dL)		104.0 [101.0,106.0]	105.0 [102.0,107.0]	103.0 [100.0,105.0]	103.0 [99.0,107.0]	<0.001
tCO2 (mEq/L)		23.9 [21.6,26.0]	24.4 [22.3,26.4]	23.4 [21.2,25.5]	25.0 [22.0,28.0]	<0.001
Magnesium (mg/dL)		0.8 [0.8,0.9]	0.8 [0.8,0.9]	1.7 [1.6,1.8]	0.8 [0.7,0.9]	<0.001
Total cholesterol (mg/dL)		160.0 [132.0,190.0]	156.0 [128.0,188.0]	160.0 [133.0,190.0]	Not available	
HDL (mg/dL)		44.0 [36.0,53.0]	42.0 [35.0,52.0]	44.0 [36.0,54.0]	44.0 [36.0,56.0]	<0.001
Triglyceride (mg/dL)		104.0 [73.0,154.0]	104.0 [72.0,155.0]	104.0 [73.0,153.0]	107.0 [76.5,169.5]	1.000
pO2 (mmHg)		84.7 [67.2,110.1]	86.6 [69.1,142.0]	83.0 [67.0,98.0]	100.0 [48.0,274.5]	<0.001
Bicarbonate (mEq/L)		23.7 [21.0,26.0]	23.9 [21.5,25.8]	23.5 [20.7,26.2]	23.0 [21.0,26.0]	<0.001
pCO2 (mmHg)		35.9 [31.5,40.0]	36.1 [32.2,39.9]	34.9 [30.6,39.0]	41.0 [36.0,47.0]	<0.001
HbA1c (%)		5.8 [5.4,6.6]	6.2 [5.6,7.3]	5.6 [5.3,6.3]	5.9 [5.4,7.1]	<0.001
pH		7.4 [7.4,7.5]	7.4 [7.4,7.5]	7.4 [7.4,7.5]	7.4 [7.3,7.4]	<0.001
ESR (mm/hr)		24.0 [10.0,52.0]	34.0 [15.0,63.0]	16.0 [6.0,36.0]	75.5 [35.2,90.8]	<0.001
CRP (mg/L)		3.6 [0.5,8.0]	1.0 [0.5,5.8]	4.0 [1.8,11.8]	69.7 [13.0,156.5]	<0.001
Procalcitonin (ng/mL)		0.1 [0.1,0.5]	0.2 [0.1,0.9]	0.1 [0.1,0.4]	Not available	
LDH (U/L)		221.0 [184.0,276.0]	194.0 [160.0,245.0]	225.0 [189.0,279.0]	245.0 [186.0,342.0]	<0.001
Lactate (mmol/L)		1.7 [1.2,2.6]	2.1 [1.5,3.2]	1.5 [1.1,2.4]	1.7 [1.2,2.5]	<0.001
CK (U/L)		89.0 [57.0,149.0]	88.0 [53.0,159.0]	89.0 [59.0,144.0]	110.0 [55.0,293.5]	<0.001
BNP (pg/mL)		94.5 [37.3,361.0]	476.0 [117.0,2250.5]	62.1 [29.5,157.9]	2856.0 [848.0,7124.0]	<0.001
AKI incidence (n)	**0**	140244 (89.1)	73600 (88.6)	61294 (89.3)	5350 (95.1)	<0.001
	**1**	17079 (10.9)	9485 (11.4)	7321 (10.7)	273 (4.9)	

Baseline characteristics of patients from three cohorts: NHIS (development), CSHH (external validation), and MIMIC-IV (external validation). Continuous variables are presented as mean ± standard deviation or median [interquartile range], and categorical variables are presented as number (percentage). Laboratory values represent the first measurement after admission. P-values were calculated using one-way ANOVA or Kruskal–Wallis test for continuous variables and chi-squared test for categorical variables, with Bonferroni correction for multiple comparisons. SMD, standardised mean difference; AKI, acute kidney injury; BMI, body-mass index; SBP, systolic blood pressure; DBP, diastolic blood pressure; ALT, alanine aminotransferase; AST, aspartate aminotransferase; ALP, alkaline phosphatase; GGT, gamma-glutamyl transferase; LDH, lactate dehydrogenase; BUN, blood urea nitrogen; BNP, brain natriuretic peptide; CK, creatine kinase; CRP, C-reactive protein; ESR, erythrocyte sedimentation rate; PT INR, prothrombin time international normalised ratio; aPTT, activated partial thromboplastin time; WBC, white blood cell; RBC, red blood cell; RDW, red cell distribution width; PDW, platelet distribution width; HbA1c, glycated haemoglobin; HDL, high-density lipoprotein.

Supplementary Table S2. Single-Point Discrimination Performance of All Models

Type	Model	PH	NHIS AUROC (95% CI)	CSHH AUROC (95% CI)	MIMIC AUROC (95% CI)
DL	ITE Transformer	0h	0.924 (0.916–0.931)	0.912 (0.909–0.914)	0.962 (0.956–0.968)
DL	ITE Transformer	48h	0.907 (0.897–0.915)	0.822 (0.816–0.827)	0.900 (0.889–0.911)
DL	ITE Transformer	72h	0.901 (0.890–0.911)	0.776 (0.771–0.782)	0.713 (0.682–0.743)
DL	LSTM-Attention	0h	0.950 (0.941–0.958)	0.864 (0.860–0.869)	0.956 (0.948–0.963)
DL	LSTM-Attention	48h	0.926 (0.916–0.935)	0.761 (0.755–0.767)	0.851 (0.838–0.864)
DL	LSTM-Attention	72h	0.928 (0.918–0.938)	0.631 (0.623–0.639)	0.599 (0.564–0.632)
DL	Masked CNN	0h	0.961 (0.955–0.967)	0.818 (0.814–0.823)	0.963 (0.957–0.967)
DL	Masked CNN	48h	0.950 (0.941–0.960)	0.793 (0.787–0.799)	0.877 (0.861–0.893)
DL	Masked CNN	72h	0.944 (0.934–0.953)	0.759 (0.753–0.766)	0.648 (0.620–0.678)
Baseline	XGBoost	0h	0.899 (0.888–0.910)	0.824 (0.819–0.829)	0.686 (0.654–0.716)
Baseline	XGBoost	48h	0.885 (0.873–0.897)	0.806 (0.801–0.811)	0.668 (0.637–0.697)
Baseline	XGBoost	72h	0.867 (0.854–0.880)	0.799 (0.793–0.805)	0.673 (0.641–0.705)
Baseline	Logistic Regression	0h	0.841 (0.825–0.855)	0.796 (0.791–0.802)	0.630 (0.595–0.663)
Baseline	Logistic Regression	48h	0.830 (0.815–0.844)	0.777 (0.771–0.782)	0.612 (0.580–0.646)
Baseline	Logistic Regression	72h	0.821 (0.806–0.836)	0.779 (0.773–0.784)	0.620 (0.584–0.654)

Supplementary Table S3. Online Simulation AUROC Trajectories (PH=0h, Matched Reference)

Type	Model	Site	72h	60h	48h	36h	24h	12h	0h	ΔAUROC
DL	ITE Transformer	NHIS	0.806	0.844	0.892	0.933	0.969	0.957	0.958	+0.153
DL	ITE Transformer	CSHH	0.765	0.805	0.870	0.901	0.954	0.959	0.947	+0.183
DL	ITE Transformer	MIMIC-IV	0.732	0.830	0.901	0.946	0.984	0.986	0.981	+0.249
DL	LSTM-Attention	NHIS	0.638	0.691	0.748	0.835	0.928	0.938	0.949	+0.311
DL	LSTM-Attention	CSHH	0.597	0.648	0.702	0.778	0.843	0.945	0.925	+0.328
DL	LSTM-Attention	MIMIC-IV	0.579	0.638	0.728	0.857	0.943	0.982	0.978	+0.398
DL	Masked CNN	NHIS	0.737	0.762	0.787	0.837	0.894	0.915	0.933	+0.196
DL	Masked CNN	CSHH	0.723	0.737	0.784	0.800	0.885	0.883	0.834	+0.111
DL	Masked CNN	MIMIC-IV	0.606	0.665	0.727	0.805	0.920	0.948	0.971	+0.366
Baseline	XGBoost	NHIS	0.782	0.798	0.824	0.844	0.876	0.888	0.902	+0.120
Baseline	XGBoost	CSHH	0.736	0.751	0.771	0.785	0.835	0.846	0.814	+0.078
Baseline	XGBoost	MIMIC-IV	0.625	0.636	0.647	0.674	0.758	0.672	0.676	+0.051
Baseline	Logistic Regression	NHIS	0.717	0.736	0.756	0.775	0.798	0.821	0.846	+0.128
Baseline	Logistic Regression	CSHH	0.695	0.709	0.730	0.743	0.787	0.812	0.770	+0.075
Baseline	Logistic Regression	MIMIC-IV	0.516	0.532	0.537	0.563	0.595	0.594	0.600	+0.084

Supplementary Table S4. Mann–Kendall Trend Test for Clinical Faithfulness (AUROC)

Model	PH	Site	τ	p-value	Sig	Sen's Slope
ITE Transformer	0h	NHIS	0.810	0.0163	Yes	0.0303
ITE Transformer	0h	CSHH	0.810	0.0163	Yes	0.0386
ITE Transformer	0h	MIMIC	0.810	0.0163	Yes	0.0413
ITE Transformer	48h	NHIS	0.524	0.1331	No	0.0196
ITE Transformer	48h	CSHH	0.714	0.0355	Yes	0.0240
ITE Transformer	48h	MIMIC	0.714	0.0355	Yes	0.0307
ITE Transformer	72h	NHIS	-0.238	0.5480	No	-0.0047
ITE Transformer	72h	CSHH	-0.238	0.5480	No	-0.0067
ITE Transformer	72h	MIMIC	0.429	0.2296	No	0.0150
LSTM-Attention	0h	NHIS	1.000	0.0027	Yes	0.0575
LSTM-Attention	0h	CSHH	0.905	0.0069	Yes	0.0616
LSTM-Attention	0h	MIMIC	0.905	0.0069	Yes	0.0806
LSTM-Attention	48h	NHIS	0.714	0.0355	Yes	0.0156
LSTM-Attention	48h	CSHH	0.619	0.0715	No	0.0386
LSTM-Attention	48h	MIMIC	0.619	0.0715	No	0.0277
LSTM-Attention	72h	NHIS	0.143	0.7639	No	0.0017
LSTM-Attention	72h	CSHH	-0.238	0.5480	No	-0.0030
LSTM-Attention	72h	MIMIC	0.238	0.5480	No	0.0024
Masked CNN	0h	NHIS	1.000	0.0027	Yes	0.0357
Masked CNN	0h	CSHH	0.714	0.0355	Yes	0.0306
Masked CNN	0h	MIMIC	1.000	0.0027	Yes	0.0665

Masked CNN	48h	NHIS	0.143	0.7639	No	0.0033
Masked CNN	48h	CSHH	-0.238	0.5480	No	-0.0033
Masked CNN	48h	MIMIC	0.619	0.0715	No	0.0293
Masked CNN	72h	NHIS	0.429	0.2296	No	0.0013
Masked CNN	72h	CSHH	0.714	0.0355	Yes	0.0027
Masked CNN	72h	MIMIC	-0.429	0.2296	No	-0.0012
XGBoost	0h	NHIS	1.000	0.0027	Yes	0.0209
XGBoost	0h	CSHH	0.810	0.0163	Yes	0.0169
XGBoost	0h	MIMIC	0.714	0.0355	Yes	0.0094
XGBoost	48h	NHIS	1.000	0.0027	Yes	0.0166
XGBoost	48h	CSHH	0.810	0.0163	Yes	0.0137
XGBoost	48h	MIMIC	0.810	0.0163	Yes	0.0113
XGBoost	72h	NHIS	1.000	0.0027	Yes	0.0154
XGBoost	72h	CSHH	0.810	0.0163	Yes	0.0120
XGBoost	72h	MIMIC	0.810	0.0163	Yes	0.0099
Logistic Regression	0h	NHIS	1.000	0.0027	Yes	0.0211
Logistic Regression	0h	CSHH	0.810	0.0163	Yes	0.0174
Logistic Regression	0h	MIMIC	0.905	0.0069	Yes	0.0156
Logistic Regression	48h	NHIS	1.000	0.0027	Yes	0.0188
Logistic Regression	48h	CSHH	0.810	0.0163	Yes	0.0148
Logistic Regression	48h	MIMIC	0.905	0.0069	Yes	0.0161
Logistic Regression	72h	NHIS	1.000	0.0027	Yes	0.0172
Logistic Regression	72h	CSHH	0.810	0.0163	Yes	0.0141
Logistic Regression	72h	MIMIC	0.905	0.0069	Yes	0.0150

Summary: PH=0h: 15/15 sig, median τ=0.905; PH=48h: 9/15 sig, median τ=0.714; PH=72h: 7/15 sig, median τ=0.429;

$\tau > 0$ = increasing AUROC toward onset. Sig = $p < 0.05$.

Supplementary Table S5. Alert Burden Analysis (PH=0h, Threshold=0.5)

Model	Site	AKI Alert%	Non-AKI Alert%	Alerts/AKI	NNE
ITE Transformer	NHIS	81.8%	7.6%	19.9	1.6
ITE Transformer	CSHH	72.4%	7.1%	10.5	1.5
ITE Transformer	MIMIC-IV	99.6%	7.4%	58.8	2.4
LSTM-Attention	NHIS	97.9%	19.9%	21.5	1.9
LSTM-Attention	CSHH	93.8%	15.0%	10.5	1.7
LSTM-Attention	MIMIC-IV	99.3%	5.5%	51.9	2.0
Masked CNN	NHIS	100.0%	100.0%	66.4	17.6
Masked CNN	CSHH	99.8%	98.6%	53.5	564
Masked CNN	MIMIC-IV	100.0%	100.0%	68.9	23.8
XGBoost	NHIS	87.9%	24.2%	47.5	4.1
XGBoost	CSHH	75.1%	22.7%	31.3	173
XGBoost	MIMIC-IV	96.9%	87.4%	59.8	19.9
Logistic Regression	NHIS	88.8%	45.7%	48.3	6.1
Logistic Regression	CSHH	80.1%	36.1%	34.0	215
Logistic Regression	MIMIC-IV	96.1%	84.7%	48.6	20.7

NNE = number needed to evaluate (1/PPV). Lower = better precision.

Supplementary Table S6. Calibration Metrics (Brier Score) Before and After Isotonic Recalibration

Type	Model	PH	NHIS Before	NHIS After	NHIS Δ%	CSHH Before	CSHH After	CSHH Δ%	MIMIC Before	MIMIC After	MIMIC Δ%
DL	ITE Transformer	0h	0.355	0.064	81.9%	0.346	0.065	81.3%	0.365	0.031	91.4%
DL	ITE Transformer	48h	0.328	0.064	80.6%	0.352	0.076	78.3%	0.428	0.040	90.7%
DL	ITE Transformer	72h	0.346	0.063	81.6%	0.401	0.084	79.0%	0.772	0.045	94.2%
DL	LSTM-Attention	0h	0.356	0.045	87.3%	0.396	0.074	81.3%	0.361	0.032	91.2%
DL	LSTM-Attention	48h	0.321	0.055	83.0%	0.345	0.084	75.6%	0.482	0.042	91.3%
DL	LSTM-Attention	72h	0.321	0.053	83.4%	0.499	0.091	81.7%	0.796	0.046	94.2%
DL	Masked CNN	0h	0.250	0.042	83.1%	0.337	0.080	76.3%	0.276	0.031	88.9%
DL	Masked CNN	48h	0.265	0.038	85.5%	0.331	0.079	76.3%	0.434	0.041	90.6%
DL	Masked CNN	72h	0.259	0.042	83.8%	0.344	0.083	75.8%	0.624	0.046	92.7%
Baseline	XGBoost	0h	0.095	0.064	32.5%	0.120	0.078	35.2%	0.426	0.045	89.4%
Baseline	XGBoost	48h	0.099	0.066	33.8%	0.122	0.079	34.9%	0.444	0.046	89.7%
Baseline	XGBoost	72h	0.107	0.069	35.6%	0.118	0.080	32.5%	0.431	0.046	89.4%
Baseline	Logistic Regression	0h	0.150	0.077	48.8%	0.163	0.081	50.3%	0.361	0.046	87.3%
Baseline	Logistic Regression	48h	0.158	0.079	50.1%	0.166	0.083	49.6%	0.365	0.046	87.4%
Baseline	Logistic Regression	72h	0.163	0.080	51.1%	0.175	0.084	52.3%	0.370	0.046	87.6%

Isotonic regression fitted using five-fold cross-validation per site. Δ% = improvement in Brier score.

Supplementary Table S7. Comparison with Prior AKI Prediction Studies

Study	Year	Model	n	Ext Val	Horizon	AUROC (ext)	Key Difference
Tomašev [7]	2019	RNN	703K	No	Cont 48h	—	No ext val; no trajectory analysis
Rank [8]	2020	RNN	15K	No	Real-time	—	Cardiac surgery only; single-centre
Koyner [34]	2018	GBM	121K	No	Static 24h	—	Traditional ML; no continuous eval
Alfieri [5]	2023	GBM	17K	3 countries	Cont hourly	0.877	No trajectory/faithfulness analysis
Cho [28]	2025	ML	Multi	2 sites	Static	Degraded	No continuous monitoring
Koyner [29]	2025	Multimodal DL	Multi	1 site	Per shift	0.842	No trajectory analysis
Zhang [10]	2025	Interp ML	178K	5 sites	Static 48h	0.74–0.85	No DL; no continuous monitoring
Present	2026	3DL+2BL	157K	2 sites, 2 countries	0/48/72h	0.91–0.96	MK-validated faithfulness; DL vs BL under continuous monitoring; alert burden

DL = deep learning; BL = baseline; GBM = gradient boosting; RNN = recurrent neural network; MK = Mann–Kendall.

Supplementary Table S8. Complete List of Input Features

x	Unit	Feature	Unit	Feature	Unit	Feature	Unit	Feature	Unit
Age	years	Uric Acid	mg/dL	Total bilirubin	mg/dL	aPTT	seconds	HDL	mg/dL
Sex	-	Sodium	mg/dL	Albumin	g/dL	BNP	pg/mL	Lactate	mmol/L
BMI	kg/m²	Potassium	mg/dL	Total Protein	g/dL	CK	U/L	pH	-
Systolic blood pressure	mmHg	Chloride	mg/dL	WBC	×10³/μL	LDH	U/L	pCO2	mmHg
Diastolic blood pressure	mmHg	Calcium	mg/dL	RBC	×10⁶/μL	CRP	mg/L	pO2	mmHg
Heart rate	/min	Magnesium	mg/dL	Haemoglobin	g/dL	ESR	mm/hr	Bicarbonate	mEq/L
Respiratory rate	/min	Phosphate	mg/dL	Haematocrit	%	Procalcitonin	ng/mL	tCO2	mEq/L
Oxygen saturation	%	ALT	U/L	Platelet count	×10³/μL	Glucose	mg/dL		
Temperature	°C	AST	U/L	RDW	%	HbA1c	%		
Creatinine	mg/dL	Alkaline phosphatase	U/L	PDW	%	Total cholesterol	mg/dL		
BUN	mg/dL	GGT	U/L	PT INR	-	Triglyceride	mg/dL		

The 51 input features used for model development, organised by category with feature name and unit. Demographics (3 features): Age, Sex, and Body-mass index. Vital signs (6 features): Systolic blood pressure, Diastolic blood pressure, Heart rate, Respiratory rate, Oxygen saturation, and Temperature. Laboratory values (42 features) organised by subcategory: kidney function (Creatinine, BUN, Uric acid), electrolytes (Sodium, Potassium, Chloride, Calcium, Magnesium, Phosphate), liver function (Albumin, Total protein, Total bilirubin, AST, ALT, Alkaline phosphatase, GGT, LDH), haematology (Haemoglobin, Haematocrit, RBC, WBC, Platelet, RDW, PDW), coagulation (PT INR, aPTT), cardiac markers (BNP, CK), metabolic markers (Glucose, HbA1c, Total cholesterol, HDL, Triglyceride), inflammatory markers (CRP, ESR, Procalcitonin), and blood gas (pH, pCO_2, pO_2, Bicarbonate, tCO_2, Lactate).

Supplementary Table S9. Missingness Rates During Hospitalisation and Measurement Frequency by Feature and Cohort

Feature	Type	NHIS Missing %	CSHH Missing %	MIMIC Missing %	NHIS Measurement Freq.	CSHH Measurement Freq.	MIMIC Measurement Freq.
Age, years	Demographic	0.0%	0.0%	0.0%	—	—	—
Sex	Demographic	0.0%	0.0%	0.0%	—	—	—
BMI, kg/m²	Demographic	0.0%	0.0%	0.0%	—	—	—
Systolic blood pressure, mmHg	Vital sign	0.0%	0.0%	0.0%	20 [12, 39] (n=83085, 100.0%)	14 [9, 27] (n=68615, 100.0%)	39 [29, 60] (n=5623, 100.0%)
Diastolic blood pressure, mmHg	Vital sign	0.0%	0.0%	0.0%	20 [12, 39] (n=83085, 100.0%)	14 [9, 27] (n=68615, 100.0%)	39 [29, 60] (n=5623, 100.0%)
Heart rate, bpm	Vital sign	0.0%	0.0%	0.0%	20 [12, 39] (n=83085, 100.0%)	15 [9, 27] (n=68615, 100.0%)	41 [30, 64] (n=5623, 100.0%)
Respiratory rate, /min	Vital sign	0.0%	0.0%	0.0%	19 [11, 37] (n=83085, 100.0%)	14 [9, 26] (n=68615, 100.0%)	39 [29, 62] (n=5623, 100.0%)
Oxygen saturation, %	Vital sign	43.8%	20.6%	0.0%	1 [0, 12] (n=46658, 56.2%)	2 [1, 6] (n=54465, 79.4%)	40 [29, 63] (n=5623, 100.0%)
Temperature, °C	Vital sign	0.0%	0.0%	0.0%	20 [11, 44] (n=83085, 100.0%)	15 [9, 29] (n=68615, 100.0%)	11 [7, 19] (n=5623, 100.0%)
WBC, ×10³/μL	Laboratory	17.3%	11.8%	3.3%	2 [1, 4] (n=68684, 82.7%)	2 [1, 4] (n=60494, 88.2%)	3 [2, 4] (n=5440, 96.7%)
Haemoglobin, g/dL	Laboratory	17.5%	12.0%	1.2%	2 [1, 4] (n=68545, 82.5%)	2 [1, 4] (n=60362, 88.0%)	3 [2, 6] (n=5558, 98.8%)
Haematocrit, %	Laboratory	17.5%	12.0%	1.1%	2 [1, 4] (n=68577, 82.5%)	2 [1, 4] (n=60357, 88.0%)	3 [2, 6] (n=5563, 98.9%)
RBC, ×10⁶/μL	Laboratory	17.5%	12.2%	1.2%	2 [1, 4] (n=68527, 82.5%)	2 [1, 4] (n=60274, 87.8%)	3 [2, 4] (n=5556, 98.8%)
RDW, %	Laboratory	17.8%	12.2%	2.1%	2 [1, 4] (n=68313, 82.2%)	2 [1, 4] (n=60239, 87.8%)	3 [2, 4] (n=5504, 97.9%)
Platelet, ×10³/μL	Laboratory	17.3%	11.9%	2.6%	4 [2, 8] (n=68682, 82.7%)	2 [1, 4] (n=60473, 88.1%)	3 [2, 4] (n=5479, 97.4%)
PDW, %	Laboratory	17.7%	69.7%	100.0%	2 [1, 4] (n=68362, 82.3%)	0 [0, 1] (n=20783, 30.3%)	Not available
PT INR	Laboratory	29.3%	26.7%	14.6%	1 [0, 2] (n=58706, 70.7%)	1 [0, 2] (n=50298, 73.3%)	2 [1, 3] (n=4801, 85.4%)
aPTT, sec	Laboratory	29.0%	26.8%	15.6%	1 [0, 2] (n=59024, 71.0%)	1 [0, 2] (n=50209, 73.2%)	2 [1, 3] (n=4744, 84.4%)
BUN, mg/dL	Laboratory	24.7%	13.7%	1.6%	1 [1, 3] (n=62545, 75.3%)	2 [1, 4] (n=59219, 86.3%)	2 [2, 4] (n=5535, 98.4%)
Creatinine, mg/dL	Laboratory	25.0%	13.7%	9.7%	2 [0, 6] (n=62294, 75.0%)	2 [1, 4] (n=59214, 86.3%)	2 [1, 4] (n=5075, 90.3%)
AST, U/L	Laboratory	24.0%	13.9%	54.7%	1 [1, 3] (n=63150, 76.0%)	2 [1, 3] (n=59057, 86.1%)	0 [0, 1] (n=2545, 45.3%)

ALT, U/L	Laboratory	24.0%	13.8%	54.0%	1 [1, 3] (n=63133, 76.0%)	2 [1, 3] (n=59143, 86.2%)	0 [0, 1] (n=2589, 46.0%)
GGT, U/L	Laboratory	84.7%	23.2%	98.9%	0 [0, 0] (n=12703, 15.3%)	1 [1, 3] (n=52700, 76.8%)	0 [0, 0] (n=63, 1.1%)
Alkaline phosphatase, U/L	Laboratory	74.5%	15.2%	52.9%	0 [0, 1] (n=21223, 25.5%)	2 [1, 3] (n=58159, 84.8%)	0 [0, 1] (n=2649, 47.1%)
Total bilirubin, mg/dL	Laboratory	36.2%	15.6%	54.0%	2 [0, 4] (n=52978, 63.8%)	2 [1, 3] (n=57922, 84.4%)	0 [0, 1] (n=2585, 46.0%)
Glucose, mg/dL	Laboratory	36.7%	14.0%	1.1%	1 [0, 2] (n=52621, 63.3%)	2 [1, 3] (n=59027, 86.0%)	3 [2, 6] (n=5559, 98.9%)
Total protein, g/dL	Laboratory	29.0%	14.0%	97.4%	1 [0, 2] (n=58997, 71.0%)	2 [1, 3] (n=59039, 86.0%)	0 [0, 0] (n=146, 2.6%)
Albumin, g/dL	Laboratory	28.7%	15.8%	67.2%	1 [0, 2] (n=59235, 71.3%)	2 [1, 3] (n=57788, 84.2%)	0 [0, 1] (n=1843, 32.8%)
Uric acid, mg/dL	Laboratory	68.1%	10.5%	98.6%	0 [0, 1] (n=26504, 31.9%)	2 [1, 3] (n=61395, 89.5%)	0 [0, 0] (n=79, 1.4%)
Calcium, mg/dL	Laboratory	70.8%	14.0%	7.9%	0 [0, 1] (n=24299, 29.2%)	2 [1, 3] (n=59023, 86.0%)	2 [1, 4] (n=5177, 92.1%)
Phosphate, mg/dL	Laboratory	71.6%	15.2%	7.5%	0 [0, 1] (n=23585, 28.4%)	2 [1, 3] (n=58187, 84.8%)	2 [1, 4] (n=5203, 92.5%)
Sodium, mEq/L	Laboratory	22.6%	14.2%	1.1%	1 [1, 4] (n=64344, 77.4%)	2 [1, 4] (n=58864, 85.8%)	3 [2, 5] (n=5563, 98.9%)
Potassium, mEq/L	Laboratory	22.6%	14.2%	1.1%	1 [1, 4] (n=64341, 77.4%)	2 [1, 4] (n=58863, 85.8%)	3 [2, 7] (n=5563, 98.9%)
Chloride, mEq/L	Laboratory	22.6%	14.2%	1.1%	1 [1, 4] (n=64334, 77.4%)	2 [1, 4] (n=58863, 85.8%)	3 [2, 5] (n=5561, 98.9%)
tCO2, mEq/L	Laboratory	54.7%	33.3%	44.3%	0 [0, 1] (n=37625, 45.3%)	1 [0, 2] (n=45772, 66.7%)	1 [0, 4] (n=3131, 55.7%)
Magnesium, mg/dL	Laboratory	78.2%	99.1%	3.7%	0 [0, 0] (n=18098, 21.8%)	0 [0, 0] (n=609, 0.9%)	2 [1, 4] (n=5415, 96.3%)
Total cholesterol, mg/dL	Laboratory	78.6%	15.1%	100.0%	0 [0, 0] (n=17761, 21.4%)	2 [1, 3] (n=58263, 84.9%)	Not available
HDL, mg/dL	Laboratory	85.0%	64.7%	94.0%	0 [0, 0] (n=12438, 15.0%)	0 [0, 1] (n=24233, 35.3%)	0 [0, 0] (n=337, 6.0%)
Triglyceride, mg/dL	Laboratory	83.1%	64.5%	90.6%	0 [0, 0] (n=14076, 16.9%)	0 [0, 1] (n=24367, 35.5%)	0 [0, 0] (n=531, 9.4%)
pO2, mmHg	Laboratory	79.1%	72.9%	44.3%	0 [0, 0] (n=17405, 20.9%)	0 [0, 1] (n=18593, 27.1%)	1 [0, 4] (n=3131, 55.7%)
Bicarbonate, mEq/L	Laboratory	79.0%	81.3%	1.1%	0 [0, 0] (n=17435, 21.0%)	0 [0, 0] (n=12813, 18.7%)	2 [2, 4] (n=5559, 98.9%)
pCO2, mmHg	Laboratory	79.0%	72.5%	44.3%	0 [0, 0] (n=17437, 21.0%)	0 [0, 1] (n=18887, 27.5%)	1 [0, 4] (n=3131, 55.7%)
HbA1c, %	Laboratory	84.9%	63.2%	92.0%	0 [0, 0] (n=12537, 15.1%)	0 [0, 1] (n=25276, 36.8%)	0 [0, 0] (n=451, 8.0%)
pH	Laboratory	91.4%	71.1%	42.0%	0 [0, 0] (n=7151, 8.6%)	0 [0, 1] (n=19851, 28.9%)	1 [0, 5] (n=3263, 58.0%)

ESR, mm/hr	Laboratory	51.9%	48.9%	99.3%	0 [0, 1] (n=39941, 48.1%)	1 [0, 1] (n=35042, 51.1%)	0 [0, 0] (n=40, 0.7%)
CRP, mg/L	Laboratory	44.1%	34.1%	96.2%	1 [0, 2] (n=46415, 55.9%)	2 [0, 3] (n=45198, 65.9%)	0 [0, 0] (n=214, 3.8%)
Procalcitonin, ng/mL	Laboratory	93.9%	82.6%	100.0%	0 [0, 0] (n=5063, 6.1%)	0 [0, 0] (n=11966, 17.4%)	Not available
LDH, U/L	Laboratory	86.5%	15.2%	67.0%	0 [0, 0] (n=11218, 13.5%)	2 [1, 3] (n=58174, 84.8%)	0 [0, 1] (n=1857, 33.0%)
Lactate, mmol/L	Laboratory	94.0%	77.3%	44.0%	0 [0, 0] (n=4975, 6.0%)	0 [0, 0] (n=15559, 22.7%)	1 [0, 2] (n=3148, 56.0%)
CK, U/L	Laboratory	76.6%	40.1%	78.7%	0 [0, 0] (n=19412, 23.4%)	1 [0, 2] (n=41121, 59.9%)	0 [0, 0] (n=1199, 21.3%)
BNP, pg/mL	Laboratory	90.9%	74.5%	97.1%	0 [0, 0] (n=7559, 9.1%)	0 [0, 1] (n=17513, 25.5%)	0 [0, 0] (n=165, 2.9%)

Missingness rate = percentage of admissions with no measurement during hospitalisation. Measurement frequency = median [Q1, Q3] number of measurements per admission (n = number of admissions with ≥1 measurement, % of total). Demographic features (Age, Sex, BMI) are recorded once at admission. N/A = variable not routinely collected in the MIMIC-IV dataset.

Supplementary Table S10. Model Performance with Reduced Feature Sets (PH=0h)

Model	Site	Full	Top50%	Δ%	Top33%	Δ%	Top20%	Δ%
ITE Transformer	NHIS	0.924	0.923	0.1%	0.922	0.2%	0.916	0.9%
ITE Transformer	CSHH	0.912	0.912	-0.0%	0.911	0.1%	0.908	0.4%
ITE Transformer	MIMIC	0.962	0.968	-0.6%	0.968	-0.6%	0.968	-0.6%
LSTM-Attention	NHIS	0.950	0.963	-1.4%	0.957	-0.7%	0.905	4.7%
LSTM-Attention	CSHH	0.864	0.869	-0.6%	0.860	0.5%	0.839	2.9%
LSTM-Attention	MIMIC	0.956	0.952	0.4%	0.954	0.2%	0.948	0.8%
Masked CNN	NHIS	0.961	0.962	-0.1%	0.957	0.4%	0.953	0.8%
Masked CNN	CSHH	0.818	0.751	8.2%	0.752	8.1%	0.746	8.8%
Masked CNN	MIMIC	0.963	0.964	-0.2%	0.962	0.1%	0.961	0.2%
XGBoost	NHIS	0.899	0.928	-3.2%	0.903	-0.4%	0.881	2.0%
XGBoost	CSHH	0.824	0.844	-2.4%	0.840	-2.0%	0.831	-0.9%
XGBoost	MIMIC	0.686	0.758	-10.5%	0.761	-10.9%	0.771	-12.4%
Logistic Regression	NHIS	0.841	0.841	0.0%	0.837	0.5%	0.817	2.9%
Logistic Regression	CSHH	0.796	0.809	-1.6%	0.799	-0.3%	0.787	1.2%
Logistic Regression	MIMIC	0.630	0.698	-10.7%	0.691	-9.7%	0.680	-7.9%

Zero-masking: features outside top-K% set to zero. Negative Δ% = improved with fewer features.

ADDITIONAL TECHNICAL NOTES

Software and Libraries

– Python: 3.13.7
– PyTorch: 2.8.0
– pandas: 2.3.0
– NumPy: 2.1.2
– scikit-learn: 1.7.0
– SHAP: 0.50 (for model interpretability; not presented in main results)

Computational Resources

– GPUs: 2× NVIDIA A4000
– RAM: 64 GB
– Training time: approximately 4–6 hours per model configuration

Reproducibility

– Random seed: 1004 (fixed for all experiments)
– Code availability: [To be provided upon publication]
– Data availability: MIMIC-IV is available through PhysioNet; Korean datasets are available upon reasonable request with appropriate ethics approval
